# Global functional profiling of human ubiquitome identifies E3 ubiquitin ligase DCST1 as a novel negative regulator of Type-I interferon signaling

**DOI:** 10.1038/srep36179

**Published:** 2016-10-26

**Authors:** Sajith Nair, Pradeep Bist, Neha Dikshit, Manoj N Krishnan

**Affiliations:** 1 Program in Emerging Infectious Diseases, Duke-NUS Medical School, 169857, Singapore

## Abstract

Type I interferon (IFN-I) mediated innate immune response controls virus infections by inducing the expression of interferon stimulated genes (ISGs). Although ubiquitination plays key roles in immune signaling regulation, a human genome-wide understanding of the role of E3 ubiquitin ligases in interferon mediated ISG induction is lacking. Here, we report a genome-wide profiling of the effect of ectopic expression of 521 E3 ubiquitin ligases and substrate recognition subunits encoded in the human genome (which constitutes 84.4% of all ubiquitination related genes encoded in the human genome, hereafter termed Human Ubiquitome) on IFNβ mediated induction of interferon stimulated DNA response element (ISRE) driven reporter activity. We identified 96 and 42 genes of the human ubiquitome as novel negative and positive regulators of interferon signaling respectively. Furthermore, we characterized DCST1 as a novel E3 ubiquitin ligase negatively regulating interferon response. Ectopic expression and gene silencing of DCST1 respectively attenuated and increased ISRE reporter activity. DCST1 regulated Type I interferon signaling by interacting with and promoting ubiquitination-mediated degradation of STAT2, an essential component of antiviral gene induction. In summary, this study provided a systems level view on the role of human ubiquitination associated genes in Type I interferon response.

The type-I interferon (IFN-I) family of cytokines (e.g., IFNα/β) is essential for controlling RNA viruses^1^,^2^,^3^. The IFN-I trigger antiviral responses by the induction of interferon induced antiviral genes (ISGs), through a complex signaling process (hereafter called IFN-I signaling pathway), beginning with the binding of IFN-I to the heterodimeric IFNα receptor IFNAR (IFNAR1 and IFNARB1)^1^,^2^,^3^,^4^,^5^. Ligand bound IFNAR will activate Janus kinase 1 (JAK1) and tyrosine kinase 2 (Tyk2), which in turn will phosphorylate the receptor subunits to recruit latent cytoplasmic transcription factors STAT1 and STAT2. JAK1 and TYK2 will phosphorylate and activate STAT1 and STAT2 respectively, leading to the formation of the ternary interferon-stimulated gene factor 3 (ISGF3) complex, composed of STAT1, STAT2 and interferon regulatory factor 9 (IRF9). Subsequently ISGF3 will migrate to the nucleus, bind to IFN-stimulated response element (ISRE) sequences, and initiate the transcription of several ISGs^6^. In addition to its anti-viral roles, IFN-I is also known to be associated with several additional biological functions such as cell proliferation, adaptive immune cell homeostasis, immune adjuvant, endotoxin shock^7^,^8^,^9^,^10^.

The regulation of IFN-I signaling by various host and pathogen factors contributes to the outcome of viral infections. While interferons serve a protective function during infection, excessive and sustained level of interferon is detrimental to the host. Therefore, the cells should tightly regulate Type-I interferon signaling; however, many of the mechanisms that optimally regulate IFN-I signaling are currently unknown.

Some of the host molecules and mechanisms governing the positive regulation of IFN-I signaling are established by previous studies. Phosphorylation controls the activation of STAT1 and STAT2, by kinases such as JAK1, TYK2, TNK1 and IKKε^11^,^12^,^13^. STAT1 function was also shown to be regulated by E3 ubiquitin ligase TRIM6 mediated phosphorylation by IKKε, hCAF1 mediated cytoplasmic retention, methylation by PRMT1 and acetylation^13^,^14^,^15^,^16^. Activated STAT proteins cooperate with cofactors p300 and CBP to initiate ISG transcription^17^. In addition, palmitoylation of IFNAR1, BRISC-SHMT2 complex mediated enhancement of IFNAR1 stability, and USP13 mediated stabilization of STAT1 protein were also implicated in IFN-I signaling^18^,^19^,^20^. Compared with our understanding of the positive regulation, less is known about the negative regulation of IFN-I signaling. The phosphatase, TC45 mediated dephosphorylation of STAT1 and Skp1-Cullin1-HOS-Roc1 ubiquitin ligase controlled downregulation of IFNAR1 are known negative regulatory mechanisms attenuating IFN-I signaling^21^,^22^.

Although ubiquitination is well known to regulate several aspects of innate immune antiviral signaling, our understanding on the role of ubiquitination related processes involved in IFN-I signaling is less comprehensive^19^,^20^,^21^,^22^,^23^. The human genome encodes for around 617 genes involved in ubiquitination, comprising both the E3 ubiquitin ligases and substrate recognition subunits, together referred to hereafter as the human ubiquitome^24^. Given their pivotal role in diverse cellular functions, it is highly likely that several members of the human ubiquitome might have unidentified roles in IFN-I signaling. However, there has not been any systematic genome-wide study to profile the role of the human ubiquitome in IFN-I signaling, through targeted experimental approaches.

We herein report the results of a systematic human genome-wide profiling of the role of the human ubiquitome in IFNβ induced IFN-I signaling, through a gain-of-function screen. We have also characterized the role of the E3 ubiquitin ligase,DCST1 as a novel negative regulator of IFN-I signaling.

## Results

### Gain-of-function screening identifies several human ubiquitome genes as regulators of type-I interferon signaling

**Figure 1:**
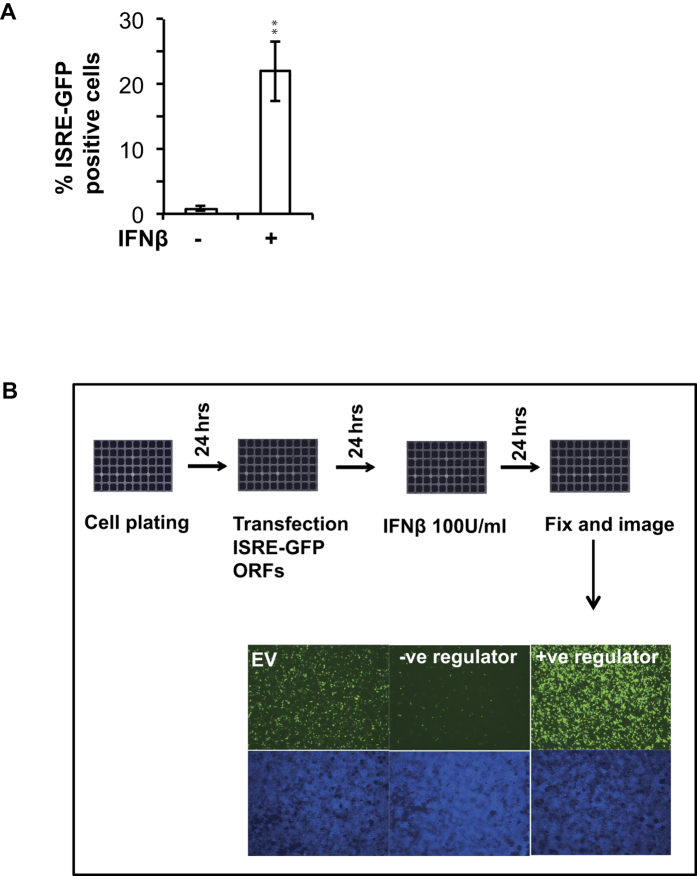
Development of a fluorescent assay to discover regulators of Type I interferon signaling pathway. (**A**) Transfected ISRE-GFP reporter expresses strongly when stimulated with 100units/ml of IFNβ, in HEK293T cells. Significance was calculated by Student’s t-test, with a p-value < 0.05 (*) was considered statistically significant (**B**) Schematic showing the organization of the gain-of-function screening experiment to discover human ubiquitome genes modulating IFNβ induced ISRE-GFP reporter activation. IFNβ, interferon beta; ORF, open reading frame; EV, empty vector.

To identify the wider role of the human ubiquitome in type-I interferon mediated ISG induction, we conducted a cell based gain-of-function screen by overexpressing various genes of the human ubiquitome and assessing their effects on ISRE driven reporter activity. The high-throughput screening assay used a green fluorescent protein (GFP) based transcriptional reporter driven by human ISRE DNA element, in human HEK293T cells stimulated with IFNβ, a widely used *in vitro* system to model IFN-I signaling. HEK293T cells transfected with 50 ng of ISRE-GFP reporter plasmid showed up to a 30-fold increase in the number of GFP expressing cells during stimulation with 100 units/ml of human recombinant IFNβ (Fig. 1A). For measuring the effect of the ubiquitome gene overexpression on ISG reporter induction, we selected 100 units/ml of IFNβ as the dose because we reasoned that this dose will be non-saturating and will provide adequate dynamic range to identify both the negative and positive regulators. The final assay involved stimulating plasmid transfected cells in 384 well plates with 100 units/ml of IFNβ for 24 hours, followed by high-content fluorescence microscopy based detection of GFP reporter signals (Fig. 1B).

Among the 617 annotated known and putative human E3 ubiquitin ligases and substrate recognition subunits present in the human genome, we assembled a library of 521 commercially available ORFs in mammalian gene expression plasmids. This constituted 84.4% of the human ubiquitome. The difference in the relative levels of GFP fluorescent cells in the ubiquitome ORF transfected cells from that of negative control (empty vector transfected) cells was measured quantitatively using the MetaXpress software (Molecular Devices Corporation). Any ubiquitome gene which upon overexpression, increased or decreased the percentage of GFP expressing cells by at least 50% of the empty vector transfected control well values were selected as positive or negative regulator genes respectively. Furthermore, to ascertain statistical robustness, the putative hit genes required that their phenotypic effects were at least 2 times the standard deviations within control wells. Wells that had a reduction in cell number of 40% or more were eliminated alluding to potential toxicity.

**Figure 2:**
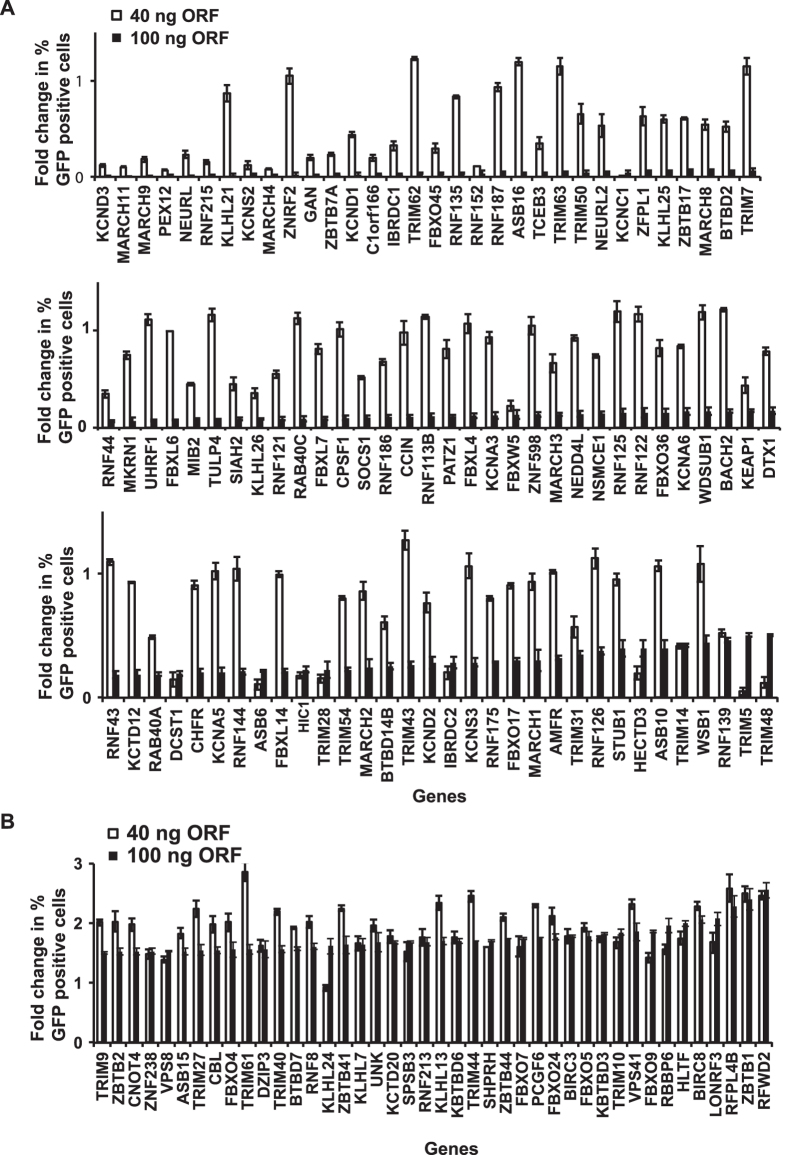
Human ubiquitome gain-of-function screen identified novel negative and positive regulators of Type I interferon pathway. (**A**) 96 ubiquitome genes served a negative regulatory effect on ISRE-GFP reporter activity upon ectopic expression. The ubiquitome genes were tested at both 40 ng and 100 ng doses. (**B**) 42 ubiquitome genes served a positive regulatory effect on ISRE-GFP reporter activity upon ectopic expression. The ubiquitome genes were tested at both 40 ng and 100 ng doses. Data is expressed as fold-change in percentage of GFP positive cells, normalized with the value corresponding to empty vector transfected negative control wells (shown as 1). Data corresponds to mean ± SD of an experiment performed in triplicates.

**Figure 3:**
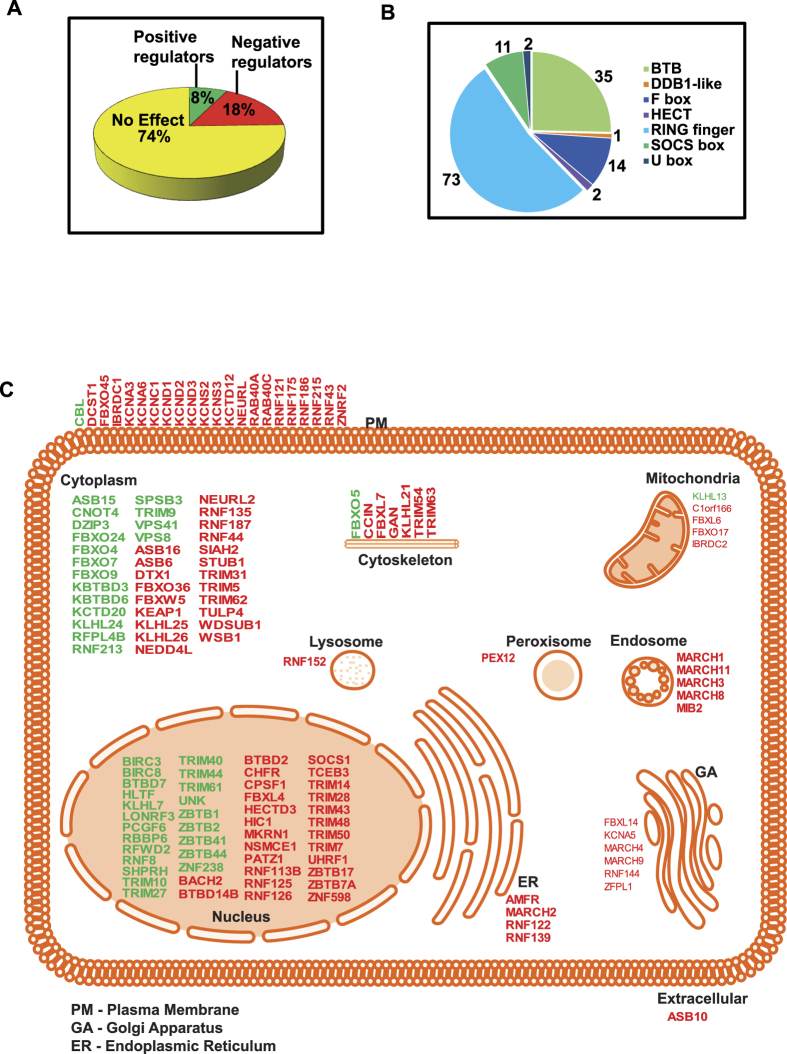
Bioinformatics analysis of IFN-I signaling modulating ubiquitome genes. (**A**) Pie chart showing the percentage of human ubiquitome genes exerting negative and positive effects on IFN-I signaling. (**B**) Pie chart showing family-wise classification of E3 ligases and substrate recognition subunits that function as modulators of IFN-I pathway. (**C**) The ubiquitome genes modulating IFN-I signaling are classified based on their known cellular location. Negative and positive regulators are respectively shown in red and green colors.

During the primary screen, we investigated the effect of over expressing two concentrations of the human ubiquitome genes (40 ng and 100 ng) on ISRE-GFP reporter activation. The screen was performed in triplicates. All of the genes that showed a 50% increase or decrease in reporter signal at 100 ng were scored as the “primary hits”. Subsequently, all primary hits that met the selection criteria were retested two more times independently in triplicates in a secondary validation screen for assessing reproducibility. After the primary discovery and secondary validation screens, we identified that ectopic expression of a total of 138 ubiquitome genes modulated ISRE reporter activity significantly, upon IFNβ stimulation (Fig. 2A,B). These included 96 negative and 42 positive regulators (Fig. 3A). Among the 96 negative regulators, only 48 genes showed significantly similar phenotype at both 40 ng and 100 ng doses of overexpressed genes (Fig. 2A). Forty out of the 42 positive regulators displayed phenotypic effect at both the tested doses (Fig. 2B). Interestingly, among the positive regulators, some of the genes showed more prominent effect at 40 ng than 100 ng.

### Bioinformatics analysis revealed that the E3 ubiquitin ligases impacting IFNβ pathway are distributed in different families

The ubiquitin ligases and their substrate recognition subunits encoded in the human genome have been classified into various groups based their structural features. All known and putative E3 ubiquitin ligases are broadly classified into four families based on their domain organization – HECT, RING finger, U-box and PHD finger. Similarly, the substrate recognition subunits primarily belong to BTB, F-box, and SOCS families.

We performed an analysis to find out if the newly identified 138 hits are distributed across many families or are enriched in certain families (Fig. 3B). Among the 138 newly identified genes, 77 were E3 ubiquitin ligases, and 61 were substrate recognition subunits. The 77 E3 ligases included 73 RING family genes that had negative and positive effects on IFN-I signaling, two HECT family members (HECTD3, NEDD4L: both negative regulators) and two U-box family E3 ligases (STUB1, WDSUB1: both negative regulators). Among the 73 RING family genes, 50 and 23 were negative and positive regulators respectively. The 61 substrate recognition subunits include 35 BTB (23 and 12 were negative and positive regulators respectively), 14 F-box (9 and 5 were negative and positive regulators respectively), 1 DDB1-like (negative regulator) and 11 SOCS (9 and 2 were negative and positive regulators respectively) family members.

We also classified the identified ubiquitome genes modulating IFN-I signaling, based on their known subcellular locations. The largest number of hits (48 genes) had known or predicted localization in the nucleus. 38 hits had known presence in the cytosol. 22 hits, including seven RING family member genes (CBL, DCST1, RNFs 121, 122, 175, 186, 215), had either validated or predicted membrane localizations. There were also genes localized in the mitochondria (5 genes), endoplasmic reticulum (4 genes), golgi (6 genes), endosome (5 genes), lysosome (1 gene), peroxisome (1 gene), cytoskeleton (7 genes) and extracellular space (1 gene). The subcellular localization of the newly identified genes whose enhanced expression affected ISG activation pathway is displayed in the schematic shown in Fig. 3C.

### Several ubiquitome genes negatively regulating IFN-I signaling are transcriptionally modulated by interferon

Negative regulation of IFN-I signaling is essential to maintain homeostasis. Although it is well known that IFN-I signaling undergoes feedback negative regulation after stimulation, the underlying mechanisms are not completely understood. Ubiquitination mediated degradation of signaling pathway proteins is a well-known mechanism employed by cells to attenuate excessive immune responses and maintain homeostasis. However, the role of E3 ubiquitin ligases in the negative regulation of IFN-I signaling is largely unknown. Using the results of the human ubiquitome over expression screen performed in this study, we therefore aimed to uncover potential novel negative regulatory ubiquitination mediated mechanisms controlling IFN-I signaling.

**Figure 4:**
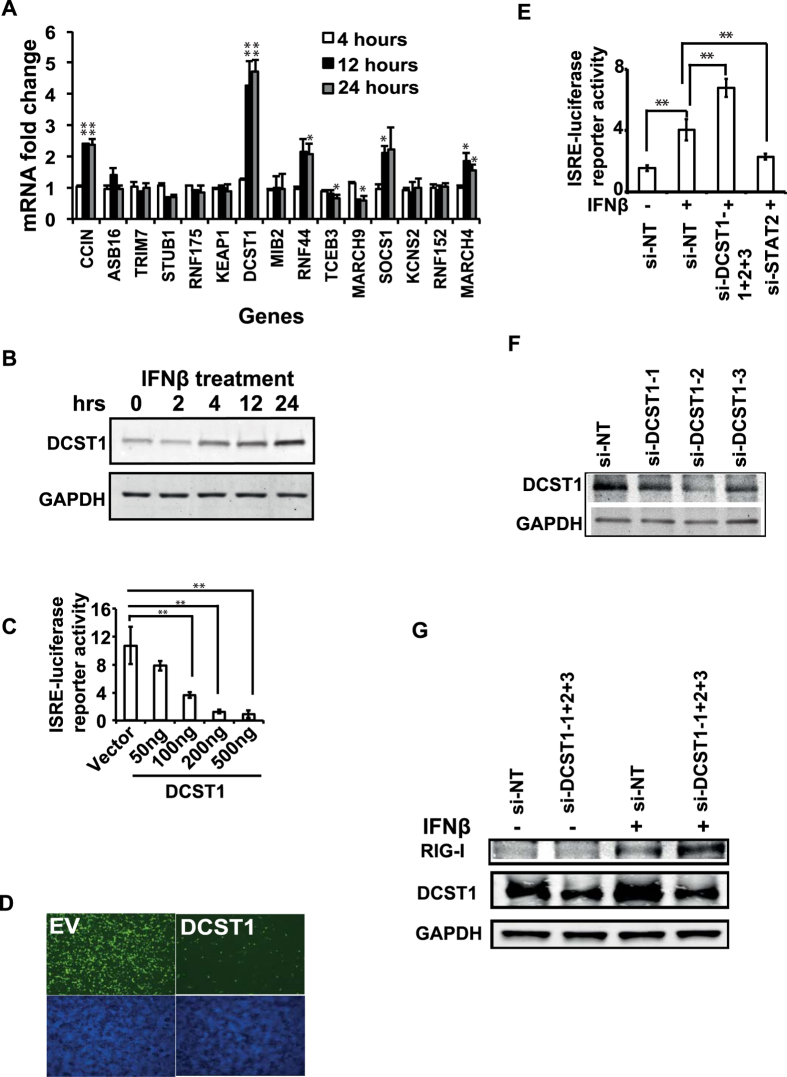
DCST1 is a negative regulator of IFN-I signaling. (**A**) IFNβ treatment modulates transcription of several human ubiquitome genes negatively regulating of IFN-I signaling. HEK293T cells were treated with human 500 units/ml IFNβ for 4, 12 and 24 hours, and gene expression was quantified by q-RTPCR. The displayed values correspond to mean ± SD from a representative experiment executed in triplicates. The given values are presented as fold change in mRNA levels relative to unstimulated sample, taken as 1. The q-RTPCR results were determined by assessing relative Ct value, using the formula Fold-change = 2^(Ct of unstimulated – Ct of stimulated) Target gene^/2^(Ct of unstimulated – Ct of stimulated) Reference gene^. We compared values for untreated samples with each one of the interferon treated samples. (**B**) Endogenous *DCST1* expression was upregulated in HEK293T cells at the protein level after IFNβ treatment. (**C**) *DCST1* overexpression attenuated IFNβ induced enhancement of ISRE-luciferase reporter activity in a dose dependent manner, HEK293T cells. The displayed values are relative ISRE-luciferase activity normalized with the internal control renila luciferase values, and correspond to mean ± SD from a representative experiment. (**D**) Overexpression of 100 ng of DCST1 attenuated IFNβ induced enhancement of ISRE-GFP reporter activity in HEK293T cells. A representative fluorescence microscopy image (4x magnification) is shown. (**E**) Silencing of *DCST1* using siRNAs enhanced ISRE-luciferase reporter activity driven by IFNβ stimulation in HEK293T cells. The displayed values are relative ISRE-luciferase activity normalized with the internal control renila luciferase values, and correspond to mean ± SD from a representative experiment. (**F**) siRNAs targeting *DCST1* efficiently knocked down DCST1 protein expression. (**G**) siRNA mediated knockdown of DCST1 enhanced the expression of ISG RIG-I upon stimulation of HEK293T cells with IFNβ for 12 hrs. Si, siRNA; NT, non-targeting negative control siRNA; hrs, hours; EV, empty vector; ng, nanogram. Significance was calculated by using ANOVA test, where *p* < 0.05 was interpreted as significant. *p < 0.05; **p < 0.01. Panels shown in Fig. 4(B,F,G) are cropped sections of full images. Supplementary Figure 1 shows uncropped images corresponding to panels shown in Fig. 4(B,F,G).

Many of the negative regulators of cellular antiviral innate immune signaling pathways are known to undergo transcriptional induction after pathway activation as a negative feedback loop. Therefore, we reasoned that bona fide negative regulators would likely be transcriptionally induced by IFN-I pathway activation by interferon. To test whether the expression of the newly identified ubiquitome genes of IFN-I pathway is sensitive to pathway activation, we selected a subset of fifteen negative regulatory genes and assessed their expression at the transcript level by q-RTPCR in HEK293T cells treated with 500 units/ml IFNβ for 6 hrs,12 hrs and 24 hrs. As shown in Fig. 4A, eight out of the fifteen studied genes showed significantly altered expression after IFNβ treatment. Among these, five genes (*CCIN, DCST1, RNF44, SOCS1, MARCH4*) were upregulated and two genes (*TCEB, MARCH9*) were downregulated. Among the negative regulatory genes induced by IFNβ, *DCST1* expression showed the highest increase upon stimulation. We determined that *DCST1* mRNA levels were upregulated by 4.2 fold (*p* 0.001) upon stimulation with IFNβ. Consistent with this, the level of DCST1 protein was also upregulated in IFNβ treated HEK293Tcells (Fig. 4B). This data suggested that *DCST1* is specifically associated with IFN-I signaling. Because *DCST1* was not previously implicated in interferon response, we chose this gene for further detailed mechanistic characterization in this study.

### Gene knockdown and ectopic expression assays identify *DCST1* as a negative regulator of IFN-I signaling

We first validated the ability of *DCST1* to suppress IFN-I signaling upon ectopic expression. For this, we transfected HEK293T cells with varying doses of *DCST1* and assessed the effect on ISRE reporter activation driven by IFNβ treatment. Ectopically expressed *DCST1* attenuated IFNβ induced enhancement of ISRE-luciferase reporter in a dose dependent manner (Fig. 4C), and also reduced ISRE-GFP reporter activity (Fig. 4D).

We next validated the endogenous role of *DCST1* in IFN-I signaling using gene silencing based complementary approach. For this, we silenced *DCST1* expression in HEK293T cells using a pool of three unique siRNAs with validated ability to repress *DCST1* mRNA levels, and assessed the impact on IFNβ induced activation of ISRE reporter activity. As shown in Fig. 4E, silencing of *DCST1* significantly enhanced the activity of ISRE luciferase reporter driven by IFNβ. *DCST1* knockdown was verified by Western blot (Fig. 4F).

A major downstream effect of stimulation of cells with type-I interferon is the induction of ISGs. As a functional proof for the negative regulatory effect of DCST1 on IFN-I signaling, we subsequently evaluated the effect of knocking down *DCST1* on the expression of RIG-I, a well-known ISG. Cells treated with siRNAs targeting *DCST1* were stimulated using IFNβ for 12 hrs, and the expression level of RIG-I was determined using Western blot. Compared to negative control siRNA treated cells, *DCST1* depleted cells showed enhanced expression of RIG-I (Fig. 4G). These experiments collectively provided evidence for the function of *DCST1* as an important negative regulator of IFN-I signaling.

### *DCST1* promotes STAT2 degradation

**Figure 5:**
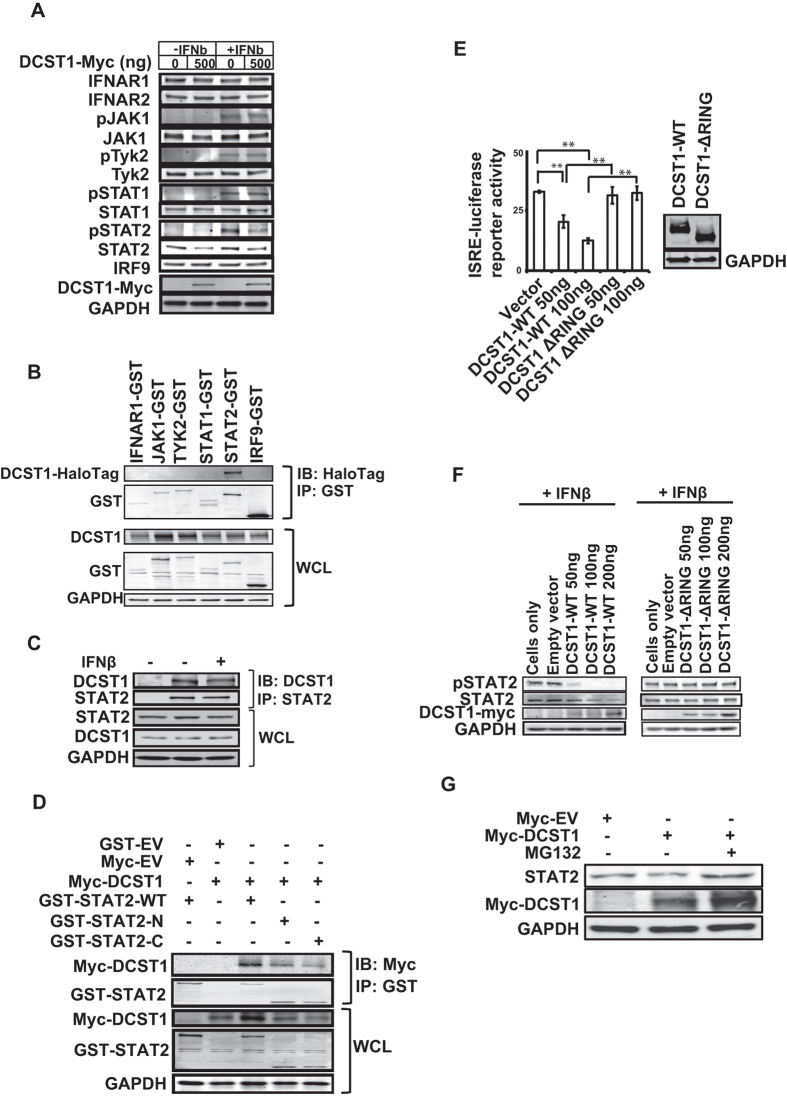
DCST1 negatively regulates the stability of STAT2. (**A**) Effect of overexpression of DCST1 (Myc tag) on the phosphorylation status (p) and stability of IFN-I pathway genes IFNAR1, IFNAR2, JAK1, TYK2, STAT1, STAT2 and IRF9 at their endogenous levels. (**B**) Ectopically expressed DCST1-Halotag interacted with ectopically expressed STAT2 (GST tag) but not other proteins of IFN-I pathway (all with GST tag). HEK293T cells were transfected with the indicated plasmids and affinity based immunoprecipitation was performed, followed by antibody based immunodetection. (**C**) Endogenous STAT2 interacted with endogenous DCST1. (**D**) DCST1 interacted with both N- and C-terminal halves of STAT2 (respectively named GST-STAT2-N and GST-STAT2-C). HEK293T cells were transfected with the indicated plasmids and affinity based immunoprecipitation was performed, followed by antibody based immunodetection. (**E**) Deletion of the RING domain abolished the ability of ectopically expressed DCST1 to suppress IFN-I pathway activation induced by IFNβ. The displayed values are relative ISRE-luciferase activity normalized with the internal control renila luciferase values, and correspond to mean ± SD from a representative experiment executed in triplicates. Inset on the right side shows that both wild type and RING deletion DCST1 were expressed at comparable levels. (**F**) RING domain deleted DCST1 was unable to promote endogenous STAT2 degradation and inhibit STAT2 phosphorylation induced by IFNβ stimulation. (**F**) Proteasome inhibitor MG132 treatment rescued the degradation of endogenous STAT2 induced by ectopically expressed DCST1 in HEK293T cells. WT, wild type; ΔRING, RING domain deletion; EV, empty vector; GST, glutathione S transferase; WCL, whole cell lysate; ng, nanogram; IB, immunoblot; IP, immunoprecipitation; Significance was calculated by using ANOVA test, where p < 0.05 was interpreted as significant. *p < 0.05; **p < 0.01. Panels shown in Fig. 5A–G are cropped sections of full images. Supplementary Figures 2–8 respectively shows uncropped images corresponding to panels shown in Fig. 5A–G.

We next investigated how *DCST1* attenuates IFN-I signaling. E3 ubiquitin ligases are well known to negatively regulate several innate immune pathways by accelerating degradation of key pathway proteins. Because overexpression of *DCST1* reduced IFN-I signaling, and *DCST1* is an E3 ubiquitin ligase, we hypothesized that it may promote degradation of one or more critical components of interferon signaling. To test this, we systematically investigated the effect of ectopic expression of *DCST1* on the stability of major known protein components of IFN-I signaling such as IFNAR1, IFNAR2, TYK2, JAK1, STAT1, STAT2 and IRF9. The experiments were performed in the presence and absence of IFNβ stimulation. The levels of each one of these proteins were measured at their endogenous levels by Western blot. It was found that enhanced expression levels of *DCST1* resulted in a prominent reduction in the protein levels of STAT2 (Fig. 5A). Accordingly, *DCST1* also reduced phosphorylated STAT2 formation induced by IFNβ. The ability of *DCST1* to degrade STAT2 levels was visible both in the presence and absence of IFNβstimulation. Ectopic expression of *DCST1* did not notably reduce the levels of any other protein of the interferon signaling cascade. This data indicated that *DCST1* negatively regulates IFN-I signaling through selectively promoting STAT2 degradation.

### DCST1 interacts with STAT2

E3 ubiquitin ligases are known to regulate targeted proteins by physically interacting with them. Because *DCST1* ectopic expression was found to degrade STAT2, we investigated whether it physically interacts with STAT2, by co-immunoprecipitation, using over expressed DCST1 and STAT2 in HEK293T cells. It was indeed found that over expressed DCST1 interacted with over expressed STAT2 (Fig. 5B). Showing specificity, DCST1 did not associate with IFNAR1, JAK1, TYK2, STAT1 or IRF9 (Fig. 5B). Additionally, we found that endogenous STAT2 interacted with endogenous DCST1 in HEK293T cells, providing further evidence that DCST1 is an authentic interacting partner of STAT2 (Fig. 5C). Interestingly, we consistently observed that stimulation with IFNβ did not increase the interaction of DCST1 with STAT2 at their endogenous levels. In the course of these experiments, we observed that efficient detection of endogenous DCST1 by Western blot required MG132 treatment, indicating the very short half-life of this protein. Similar properties were previously reported in the case of many E3 ubiquitin ligases^23^. We also went on to determine the regions of STAT2 involved in binding to DCST1. For this, we truncated STAT2 in the exact middle to generate plasmids expressing Glutathione S transferase (GST) tagged N-terminal and C-terminal halves of STAT2, and assessed their ability to interact with Myc-tagged DCST1 upon ectopic expression in HEK293T cells. As given in Fig. 5D, It was found that DCST1 was able to interact with both the N-and C-terminal halves of STAT2, indicating that a wider region of STAT2 is involved in the interaction with DCST1.

### DCST1 promotes STAT2 degradation in RING domain dependent manner

Many E3 ubiquitin ligases are known to promote degradation of proteins through ubiquitination, using their catalytic activity. Therefore, we investigated if DCST1 degraded STAT2 through its catalytic activity. For this, we deleted the catalytic RING domain of DCST1, over expressed the catalytically inactive DCST1 in HEK293T cells, and assessed its effect on IFNβ mediated ISRE reporter activity. We observed that the ectopically expressed RING domain deleted DCST1 (DCST1 ΔRING) was unable to suppress ISRE reporter activity, unlike the wild type DCST1 (Fig. 5E). Both wild type DCST1 and DCST1 ΔRING mutant were expressed at comparable levels (Fig. 5E, side panel). We also investigated if the RING domain was needed for DCST1 to promote STAT2 degradation. It was found that RING domain deficient DCST1 was unable to promote degradation of endogenous STAT2 (Fig. 5F). Similarly, wild type but not DCST1 ΔRING, suppressed STAT2 phosphorylation (Fig. 5F). Both wild type and RING deleted DCST1 proteins were expressed at comparable levels (Fig. 5F). These data demonstrated that the catalytic activity was essential for DCST1 to induce STAT2 degradation. Several E3 ubiquitin ligases are known to promote degradation of target proteins through the proteasome, a process that could be reversed by inhibiting proteasome. Our results also showed that treating cells with proteasome inhibitor MG132 attenuated the ability of ectopically expressed DCST1 to degrade endogenous STAT2 (Fig. 5G).

### DCST1 promotes ubiquitination of STAT2

**Figure 6:**
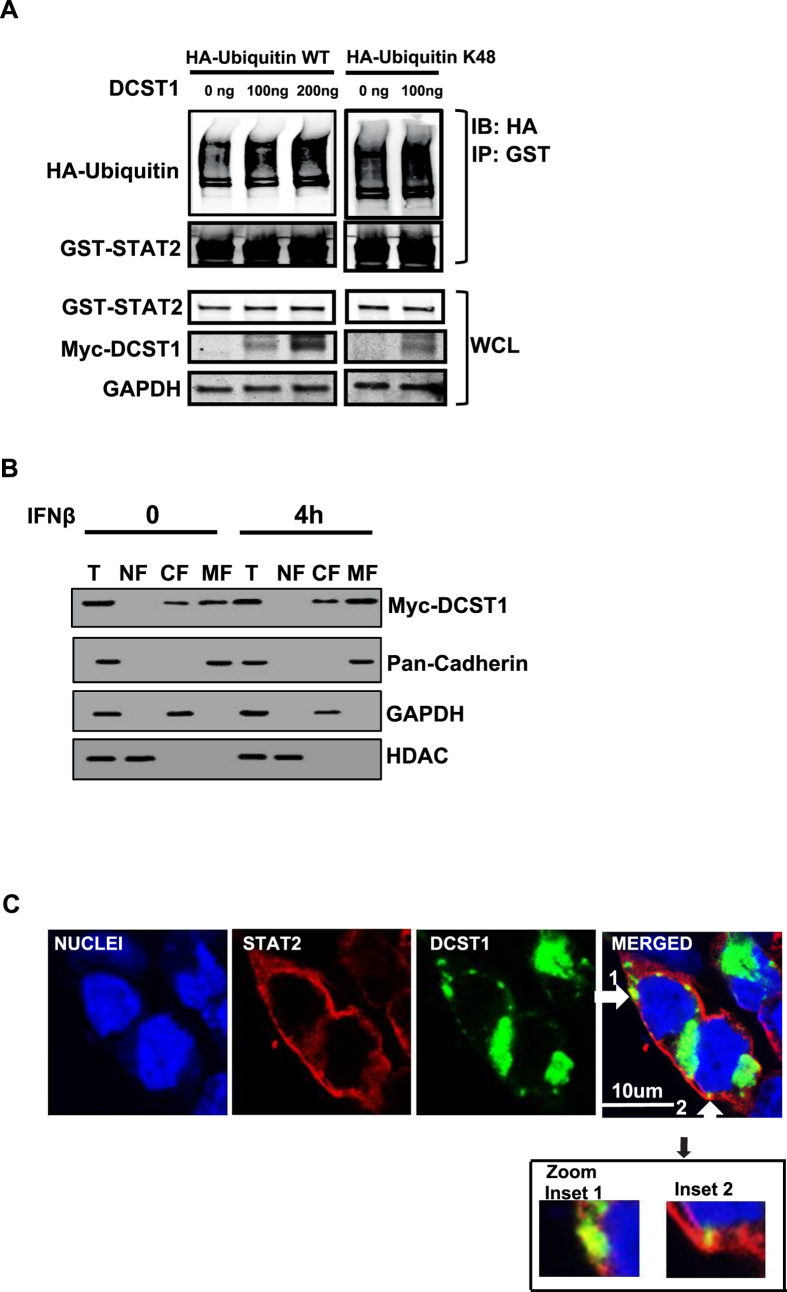
DCST1 mediated ubiquitiation and subcellular localization. (**A**) DCST1 promotes ubiquitination of STAT2. Ectopically expressed GST-STAT2 showed enhanced ubiquitination with both wildtype (WT) and K48-only mutant (K48) ubiquitins when co-expressed along with Myc-DCST1. HA-tagged Ubiquitin wildtype and K48-only mutant ubiquitins were co-transfected to detect ubiquitination. (**B**) Centrifugation based cell fractionation identified DCST1 (Myc tag) localization in both plasma membrane and cytosolic fractions by Western blot. Antibodies detecting pan-Cadherin, GAPDH and HDAC respectively were used as markers for plasma membrane, cytosol and nuclear compartments. T, total lysate; NF, nuclear fraction; CF, cytosol fraction; MF, plasma membrane fraction. (**C**) Colocalization of DCST1 and STAT2. Over expressed DCST1 (GFP tag, green) showed localization in both cell periphery and intracellular locations, as determined by fluorescent confocal microscopy. Immuno-staining for co-expressed STAT2-FLAG (red) revealed co-localization of STAT2 and DCST1 (shown with white arrows). Inset 1 and 2 shows enlarged views of colocalization spots. GST, glutathione S transferase; WCL, whole cell lysate; ng, nanogram; IB, immunoblot; IP, immunoprecipitation; DAPI, (4′,6-diamidino-2- phenylindole) fluorescent nuclear stain; GFP, green fluorescent protein. Panels shown in Fig. 6A,B are cropped sections of full images. Supplementary Figures 9 and 10 respectively shows uncropped images corresponding to panels shown in Fig. 6A,B.

Since DCST1 was determined to regulate STAT2 protein levels and IFN-I signaling pathway through its RING domain, we next examined whether DCST1 regulates IFN-I signaling through directly promoting ubiquitination of STAT2. For determining this, we transfected DCST1 into HEK293T cells, and assessed the ubiquitination of STAT2 by Western blot. It was observed that ectopic expression of DCST1 enhanced STAT2 ubiquitination in a dose dependent manner (Fig. 6A). K48 conjugated ubiquitination has widely been known as the key signal for ubiquitination based degradation of target proteins. Therefore we determined whether DCST1 could induce K48 conjugation of ubiquitin to STAT2. To test this, we transfected GST-STAT2, Myc-DCST1 and HA-tagged ubiquitin containing only K48 into HEK293T cells, followed by Western blot based monitoring of ubiquitination of STAT2. Indeed it was observed that DCST1 promoted conjugation of only K48 lysine containing ubiquitin to STAT2, indicating that it promotes K48 ubiquitination of STAT2 (Fig. 6A). These data provided further evidence that DCST1 regulated STAT2 levels through ubiquitination.

### DCST1 co-localizes with STAT2

DCST1 protein has seven transmembrane segments and is predicted to have plasma membrane localization. We first determined the subcellular localization of DCST1 using a highly sensitive centrifugation based subcellular fractionation approach. The proteins (DCST1, cellular markers) in various cellular fractions were detected using Western blot. Consistent with the bioinformatics predictions, DCST1 was found to be present predominantly on the plasma membrane; however, a small quantity of DCST1 was also found intracellularly (Fig. 6B). Next, we investigated where in the cell DCST1 and STAT2 would interact, using fluorescence microscopy. For this, green fluorescent protein tagged DSCT1 (DCST1-GFP) and FLAG tagged STAT2 were expressed in HEK293T cells for 24 hours, and imaged using fluorescence microscopy. As shown in Fig. 6C, we saw green signal intensity corresponding to DCST1 both in the cell periphery and within the cells. The presence of DCST1-GFP signal at both the cell periphery and intracellular locations was consistent with the DCST1 distribution results obtained from subcellular fractionation based experiments. Interestingly, we consistently observed that patches of DCST1 co-localized with STAT2. These data demonstrated that DCST1 and STAT2 co-localized within the cell.

## Discussion

Optimal regulation of type I interferon (IFN-I) response and IFN-I induced antiviral mechanisms are essential for both infection control and maintaining immune homeostasis. In this study, we determined the effect of enhancing the cellular levels of 84.4% of the ubiquitination related genes encoded in the human genome (termed the human ubiquitome) on IFN-I mediated transcription processes. We identified that the elevated expression of 96 and 42 genes of the human ubiquitome respectively reduced and increased IFN-I responsive transcription reporter assay. While some of these phenotypes could be due to gain-of-function induced pleiotropic effects, complementary approaches such as gene silencing are needed to further ascertain the specificity of their association with ISG transcription. Furthermore, we determined that the E3 ubiquitin ligase DCST1, one of the newly identified regulators of IFN-I signaling, negatively regulates antiviral response pathway by promoting STAT2 degradation.

We compared our results with previous studies involving discovery of regulators of IFN-I signaling pathway to find out synergistic findings. An earlier study investigated the effect of over expression of 78 TRIM family of ubiquitin ligases on RIG-I CARD domain induced activation of ISRE-promoter and identified that TRIM9, TRIM5, TRIM50, TRIM66 and TRIM67 positively regulate ISRE reporter activity^23^. Among these, Trim9 was scored as a positive regulator of ISRE reporter activity in our screen also. Although TRIM67 was not included in the final list of hit genes scored in this study as immune modulators, our primary screen identified that over expressed TRIM67 increased ISRE reporter activity but fell below our stringent hit selection criteria. TRIM66 was not tested in this study, as our expression plasmid collection did not include this gene. Unlike the phenotype observed in the previous report, our study identified a negative regulatory role for TRIM5 and TRIM50. It should be highlighted that the previous study did not use any prior stimulation with IFNβ, which could potentially contribute to the observed differences between the two screens. The ubiquitin ligase SIAH, identified in our study as a negative regulator, was previously shown to degrade TYK2^25^. The re-identification of previously known regulators of IFN-I signaling SIAH2 and TRIM9 in our screen confirm that the approach we undertook was reliable.

The bioinformatics analysis highlighted that the hit genes were distributed across many families of ubiquitin ligases and adaptors. Several of these newly identified ubiquitome components regulating IFN-I pathway were previously implicated in immune response, and we here discuss some of these genes. Previously identified negative (RNF125) and positive (RNF135, TRIM14) regulators of RIG-I mediated IFN-I transcription were found to suppress IFN-I signaling in our study^26^,^27^,^28^. Similarly, RNF121, a previously reported positive regulator of innate immune receptor dependent NFkB activation, was found to attenuate IFN-I signaling upon overexpression in this study^29^. IFN-I signaling suppressor STUB1 was previously identified to promote TLR4 and TLR9 signaling^30^. A known negative regulator of TLR3 induced interferon transcription, TRIM38, also acted similarly on IFN-I signaling^31^. Overall these evidences further display a wider role for the human ubiquitome genes in the antiviral and related innate immune pathways.

We identified and characterized DCST1 as a major ubiquitin ligase negatively regulating IFN-I signaling in this study. There has not been any previous report on the role of DCST1 in any aspect of cellular functioning. In our study, DCST1 was found to interact with and promote ubiquitination of STAT2, leading to reduced STAT2 expression and attenuated activation of the ISG induction pathway. DCST1 expression was found to be upregulated during IFN-I signaling activation, most likely as a feedback negative regulatory mechanism to attenuate the effects of persistent interferon response. Interestingly, bioinformatics analysis revealed that DCST1 promoter region contains binding sites for STAT2 and IRF9 (Krishnan at al, unpublished results). An intriguing aspect of our results pertains to the relative insensitivity of the interaction of DCST1 with STAT2 on interferon stimulation. This indicates that DCST1 likely has a key role in maintaining basal STAT2 levels also, to prevent enhanced basal interferon response.

In conclusion, the findings of this study generated a global view of the role of human ubiquitome in type-I interferon mediated downstream signaling leading to ISG induction. In addition, we have also identified DCST1 as a new E3 ubiquitin ligase that negatively regulates interferon response and maintains homeostasis. Future efforts in developing small molecule inhibitors of DCST1 may make this E3 ubiquitin ligase as a pharmacological target to enhance interferon response.

## Materials and Methods

### Human Ubiquitination associated gene library

Based on a previous human genome-wide annotation study^24^ of 617 genes involved in the ubiquitination process (including E3 ubiquitin ligases and substrate recognition subunits), we assembled an expression plasmid library of 521 of those genes from commercial ORF libraries (tested genes are listed in Supplementary Table 1). The genes were expressed from either the lentivirus pLX304 vector (Broad Institute) or the non-lentiviral pHTC HaloTag CMV-neo vector (Promega/Kasuza) or non-lentiviral pCMV sports6 vector (Dana Farber Cancer Institute).

### Cells, antibodies, ligands, transfection reagents and virus

HEK 293T (#ATCC CRL-11268) was purchased from ATCC. Antibodies for the proteins IFNAR1 (#sc-7391), pIFNAR1 (#sc-13114-R), IFNAR2 (#sc-704), JAK1 (#sc-7228), pJAK1 (#sc-101716), Tyk2 (#sc-169), pTyk2 (#sc-11763), STAT1 (#sc-271661), pSTAT1 (#sc-135648), STAT2 (#sc-476), pSTAT2 (sc-21689), IRF9 (#sc-496), DCST1 (#sc-133501), GST (#sc-459) and anti-V5 fusion proteins (#sc-83849) were all purchased from Santa Cruz; GAPDH antibody (cat# G9545) was purchased from Sigma; anti-HALO (# G9281) was purchased from Promega. Recombinant human Interferon beta (IFNβ) 1b, expressed in *E.coli* was purchased from pbl assay science (#11420-1). Polyethylenimine “Max”, (Mw 40,000) – High Potency Linear PEI (PEI) was purchased from Polysciences (# 24765-2).

### Type I interferon pathway ISRE reporter activation assay and ORF gain-of-function screening

A lentiviral reporter plasmid containing ISRE DNA element driven green fluorescent protein (GFP) (System Biosciences, cat# TR016PA-1) was used for monitoring the activation of the type I interferon pathway. Cells transfected with the reporter plasmid was stimulated with 100 units/ml of IFNβ for 24 hours, fixed and imaged to determine the GFP signal. A library of 521 human E3 ubiquitin ligase expressing plasmids were screened for their ability to modulate the levels of the ISRE-GFP reporter activity by ectopic expression, in a quantitative fluorescence microscopy based screening approach. Briefly, 7500 HEK-293T cells (in 30 ul DMEM with 10% fetal bovine serum) plated per well in a 384-well imaging compatible plate (Corning #3712) was co-transfected with either 40 or 100 ng of the ORFs and the ISRE-GFP reporter plasmid (50 ng) using the transfection reagent PEI. 24 hours post-transfection, the cells were stimulated with recombinant human IFNβ at a final concentration of 100 Units/ml. 24 hours after IFNβ stimulation, the cells were fixed using 3% paraformaldehyde, washed with phosphate buffer saline and nuclear stained with DAPI. The plates were imaged using high content fluorescence microscope (ImageXpress Micro, Molecular Devices Corporation) at 4x magnification for each well. The GFP signal was quantified by automated scoring of the number of GFP positive cells in each image using the software MetaXpress (Molecular Devices Corporation).

### Luciferase Reporter assay

HEK-293T cells were transfected with an ISRE-promoter driven firefly luciferase reporter (Biomyx, Cat# pHTS-ISRE) and a constitutively transcribed Renilla luciferase reporter (Promega, Cat#E2241) using the transfection reagent PEI. At 24 hours, the cells were stimulated with recombinant human IFNβ for 24 hours. Following which the luciferase activity measurement was performed (Promega cat# E2920), according to manufacturer’s instructions.

### Bioinformatics analysis

Subcellular localization information was gathered from various databases such as Genecards and LOCATE.

### RNA interference

siRNAs targeting DCST1 was purchased from Dharmacon, and transfected using the lipid Dharmafect 1 (Dharmacon cat#T-1001-03). DCST1 targeting siRNA sequences were: si-DCST1-1: Sense: 5′ U.G.U.A.U.G.A.G.C.U.G.A.A.G.A.C.C.A.A.U.U 3′; Antisense: 5′ U.U.G.G.U.C.U.U.C.A.G.C.U.C.A.U.A.C.A.U.U 3′; si-DCST1-2: Sense: 5′ C.C.U.C.A.G.A.G.A.C.A.G.U.G.A.U.G.G.A.U.U 3′; Antisense: 5′ U.C.C.A.U.C.A.C.U.G.U.C.U.C.U.G.A.G.G.U.U 3′; si-DCST1-3: Sense: 5′ U.A.A.A.A.U.G.C.C.C.U.G.U.A.C.G.C.U.U.U.U 3′; Antisense: 5′ 5′-P.A.A.G.C.G.U.A.C.A.G.G.G.C.A.U.U.U.U.A.U.U 3′. A pool of si-DCST1-1, si-DCST1-2 and si-DCST1-3 was used for all of the experiments described in the manuscript.

### Western blotting

The proteins were transfected in 3 × 10^5^ HEK-293T cells per well in a 24-well plate, using the transfection reagent PEI. 24 hours post-transfection, the cells were stimulated with recombinant human IFNβ at a final concentration of 500 Units/ml, following which the cells were harvested in RIPA buffer (50 mM Tris-HCl, 150 mM NaCl, 1% NP-40, 0.1% SDS and protease and phosphatase inhibitor cocktail (ThermoFischer, Cat. # 1861281)). 50 ug of clarified total cellular lysate was loaded per well to be separated on an SDS-PAGE followed by western blotting on nitrocellulose membrane. Using primary antibodies, individual proteins were detected, through the infrared-based detection system (Licor), with the IRDye 800 CW and 680 RD coupled secondary antibodies. Western blots in Fig. 6B were probed using HRP conjugated secondary antibody, and detected using ECL detection kit (Amersham).

### Co-immuno precipitation and ubiquitination assays

For the immunoprecipitation experiments, up to 2 × 10^6^ HEK293T cells propagated in 6-well plates were lysed in lysis buffer (150 mM NaCl, 1% NP40 and 50 mM Tris-Cl (pH 7.0) with protease inhibitors). The clarified supernatants (by centrifuging for 15 min at 12000 × g) were incubated overnight at 4 °C with primary antibodies (2 μg), and the antibody-antigen complexes were separated using protein G Agarose (santa cruz Biotech). After extensive washing with the lysis buffer, the proteins were separated by electrophoresis and potential protein interactions were investigated by Western blot. The proteins were detected using infrared-based detection system (Licor), with the IRDye 800CW and 680RD coupled secondary antibodies.

For ubiquitination assays, 1 × 10^7^ HEK293T cells propagated in 100 cm plates were transfected with GST-STAT, Myc-DCST1, HA-Ubiquiitn (WT or K48 mutant) for 24 hrs, and lysed in lysis buffer (150 mM NaCl, 1% NP40 and 50 mM Tris-Cl (pH 7.0). The samples were processed as described above, immuno-precipitations were performed using GST-agarose beads (Santa Cruz Biotechnology).

### Gene expression analysis

Total cellular RNA from HEK-293T cells was isolated using RNeasy kit (QIAGEN #74106). 1 ug of RNA was used for cDNA synthesis using iScript cDNA synthesis kit (BIO-RAD #170-8891). Quantitative real-time PCR was performed in C1000 thermal cycler (BIO-RAD) using the iQ SYBR Green Supermix (BIO-RAD #170-8880) to measure the transcription levels of the target genes. GAPDH was used as the constitutively expressing internal control gene. The fold-change in gene expression was calculated using the equation: Fold-change = 2^(Ct of unstimulated – Ct of stimulated) Target gene^/ 2^(Ct of unstimulated – Ct of stimulated) Reference gene^. Supplementary Table 2 lists primers used for quantitative real-time PCR.

### Subcellular fractionation

HEK293T cells lysate (in lysis buffer 10 mM Tris-HCl pH 7.5, 10 mM KCl, 0.5 mM EGTA, 1.5 mM MgCl2 and protease inhibitor cocktail) was centrifuged at 1000 g for 5 min. The resulting supernatant was subsequently centrifuged at 5000 g for 10 min. To harvest cytosolic fraction, the resulting supernatant was again centrifuged at 100,000 g for 1 h, and the supernatant was saved as cytoplasm. The pellet fraction contains plasma membrane.

### Statistics

Significance was determined by either ANOVA with Tukey’s post-hoc comparison (Figs 4 A,C,E and 5E), or two tailed Student’s t-test (Fig. 1A). *p*-values < 0.05 were considered statistically significant. *p-value < 0.05; **p-value < 0.01.

## Additional Information

**How to cite this article**: Nair, S. *et al*. Global functional profiling of human ubiquitome identifies E3 ubiquitin ligase DCST1 as a novel negative regulator of Type-I interferon signaling. *Sci. Rep.* **6**, 36179; doi: 10.1038/srep36179 (2016).

**Publisher’s note**: Springer Nature remains neutral with regard to jurisdictional claims in published maps and institutional affiliations.

## Supplementary Material

Supplementary Information

## References

[b1] Borden E. C. . Interferons at age 50: past, current and future impact on biomedicine. Nature reviews. Drug discovery 6, 975–990, doi: 10.1038/nrd2422 (2007). 18049472 PMC7097588

[b2] Kumagai Y. & Akira S. Identification and functions of pattern-recognition receptors. The Journal of allergy and clinical immunology 125, 985–992, doi: 10.1016/j.jaci.2010.01.058 (2010). 20392481

[b3] Mogensen K. E., Lewerenz M., Reboul J., Lutfalla G. & Uze G. The type I interferon receptor: structure, function, and evolution of a family business. Journal of interferon & cytokine research: the official journal of the International Society for Interferon and Cytokine Research 19, 1069–1098 (1999). 10.1089/107999099313019 10547147

[b4] Schindler C., Fu X. Y., Improta T., Aebersold R. & Darnell J. E. Jr. Proteins of transcription factor ISGF-3: one gene encodes the 91-and 84-kDa ISGF-3 proteins that are activated by interferon alpha. Proceedings of the National Academy of Sciences of the United States of America 89, 7836–7839 (1992). 1502203 10.1073/pnas.89.16.7836 PMC49806

[b5] Schindler C., Shuai K., Prezioso V. R. & Darnell J. E. Jr. Interferon-dependent tyrosine phosphorylation of a latent cytoplasmic transcription factor. Science 257, 809–813 (1992). 1496401 10.1126/science.1496401

[b6] Schoggins J. W. & Rice C. M. Interferon-stimulated genes and their antiviral effector functions. Current opinion in virology 1, 519–525, doi: 10.1016/j.coviro.2011.10.008 (2011). 22328912 PMC3274382

[b7] Apelbaum A., Yarden G., Warszawski S., Harari D. & Schreiber G. Type I interferons induce apoptosis by balancing cFLIP and caspase-8 independent of death ligands. Molecular and cellular biology 33, 800–814, doi: 10.1128/MCB.01430-12 (2013). 23230268 PMC3571350

[b8] Karaghiosoff M. . Central role for type I interferons and Tyk2 in lipopolysaccharide-induced endotoxin shock. Nature immunology 4, 471–477, doi: 10.1038/ni910 (2003). 12679810

[b9] Mattei F., Schiavoni G. & Tough D. F. Regulation of immune cell homeostasis by type I interferons. Cytokine & growth factor reviews 21, 227–236, doi: 10.1016/j.cytogfr.2010.05.002 (2010). 20627800

[b10] Prchal M. . Type I interferons as mediators of immune adjuvants for T- and B cell-dependent acquired immunity. Vaccine 27 Suppl 6, G17–G20, doi: 10.1016/j.vaccine.2009.10.016 (2009). 20006134

[b11] Krishnan K., Pine R. & Krolewski J. J. Kinase-deficient forms of Jak1 and Tyk2 inhibit interferon alpha signaling in a dominant manner. European journal of biochemistry / FEBS 247, 298–305 (1997). 10.1111/j.1432-1033.1997.00298.x 9249040

[b12] Ooi E. L. . Novel antiviral host factor, TNK1, regulates IFN signaling through serine phosphorylation of STAT1. Proceedings of the National Academy of Sciences of the United States of America 111, 1909–1914, doi: 10.1073/pnas.1314268111 (2014). 24449862 PMC3918791

[b13] Rajsbaum R. . Unanchored K48-linked polyubiquitin synthesized by the E3-ubiquitin ligase TRIM6 stimulates the interferon-IKKepsilon kinase-mediated antiviral response. Immunity 40, 880–895, doi: 10.1016/j.immuni.2014.04.018 (2014). 24882218 PMC4114019

[b14] Altschuler L., Wook J. O., Gurari D., Chebath J. & Revel M. Involvement of receptor-bound protein methyltransferase PRMT1 in antiviral and antiproliferative effects of type I interferons. Journal of interferon & cytokine research: the official journal of the International Society for Interferon and Cytokine Research 19, 189–195, doi: 10.1089/107999099314333 (1999). 10090404

[b15] Chapat C. . hCAF1/CNOT7 regulates interferon signalling by targeting STAT1. The EMBO journal 32, 688–700, doi: 10.1038/emboj.2013.11 (2013). 23386060 PMC3594750

[b16] Kramer O. H. & Heinzel T. Phosphorylation-acetylation switch in the regulation of STAT1 signaling. Molecular and cellular endocrinology 315, 40–48, doi: 10.1016/j.mce.2009.10.007 (2010). 19879327

[b17] Bhattacharya S. . Cooperation of Stat2 and p300/CBP in signalling induced by interferon-alpha. Nature 383, 344–347, doi: 10.1038/383344a0 (1996). 8848048

[b18] Claudinon J. . Palmitoylation of interferon-alpha (IFN-alpha) receptor subunit IFNAR1 is required for the activation of Stat1 and Stat2 by IFN-alpha. The Journal of biological chemistry 284, 24328–24340, doi: 10.1074/jbc.M109.021915 (2009). 19561067 PMC2782026

[b19] Yeh H. M. . Ubiquitin-specific protease 13 regulates IFN signaling by stabilizing STAT1. Journal of immunology 191, 3328–3336, doi: 10.4049/jimmunol.1300225 (2013). 23940278

[b20] Zheng H. . A BRISC-SHMT complex deubiquitinates IFNAR1 and regulates interferon responses. Cell reports 5, 180–193, doi: 10.1016/j.celrep.2013.08.025 (2013). 24075985 PMC3813903

[b21] Kumar K. G. . SCF(HOS) ubiquitin ligase mediates the ligand-induced down-regulation of the interferon-alpha receptor. The EMBO journal 22, 5480–5490, doi: 10.1093/emboj/cdg524 (2003). 14532120 PMC213778

[b22] ten Hoeve J. . Identification of a nuclear Stat1 protein tyrosine phosphatase. Molecular and cellular biology 22, 5662–5668 (2002). 12138178 10.1128/MCB.22.16.5662-5668.2002 PMC133976

[b23] Versteeg G. A. . The E3-ligase TRIM family of proteins regulates signaling pathways triggered by innate immune pattern-recognition receptors. Immunity 38, 384–398, doi: 10.1016/j.immuni.2012.11.013 (2013). 23438823 PMC3584420

[b24] Li W. . Genome-wide and functional annotation of human E3 ubiquitin ligases identifies MULAN, a mitochondrial E3 that regulates the organelle’s dynamics and signaling. PloS one 3, e1487, doi: 10.1371/journal.pone.0001487 (2008). 18213395 PMC2198940

[b25] Muller S. . SIAH2 antagonizes TYK2-STAT3 signaling in lung carcinoma cells. Oncotarget 5, 3184–3196 (2014). 24833526 10.18632/oncotarget.1899 PMC4102802

[b26] Arimoto K. . Negative regulation of the RIG-I signaling by the ubiquitin ligase RNF125. Proceedings of the National Academy of Sciences of the United States of America 104, 7500–7505, doi: 10.1073/pnas.0611551104 (2007). 17460044 PMC1863485

[b27] Oshiumi H., Matsumoto M., Hatakeyama S. & Seya T. Riplet/RNF135, a RING finger protein, ubiquitinates RIG-I to promote interferon-beta induction during the early phase of viral infection. The Journal of biological chemistry 284, 807–817, doi: 10.1074/jbc.M804259200 (2009). 19017631

[b28] van der Lee R. . Integrative Genomics-Based Discovery of Novel Regulators of the Innate Antiviral Response. PLoS computational biology 11, e1004553, doi: 10.1371/journal.pcbi.1004553 (2015). 26485378 PMC4618338

[b29] Zemirli N., Pourcelot M., Dogan N., Vazquez A. & Arnoult D. The E3 ubiquitin ligase RNF121 is a positive regulator of NF-kappaB activation. Cell communication and signaling: CCS 12, 72, doi: 10.1186/s12964-014-0072-8 (2014). 25388546 PMC4232610

[b30] Yang M. . E3 ubiquitin ligase CHIP facilitates Toll-like receptor signaling by recruiting and polyubiquitinating Src and atypical PKC{zeta}. The Journal of experimental medicine 208, 2099–2112, doi: 10.1084/jem.20102667 (2011). 21911421 PMC3182058

[b31] Xue Q. . TRIM38 negatively regulates TLR3-mediated IFN-beta signaling by targeting TRIF for degradation. PloS one 7, e46825, doi: 10.1371/journal.pone.0046825 (2012). 23056470 PMC3466175

