# Supplementary material for: Global functional profiling of human ubiquitome identifies E3 ubiquitin ligase DCST1 as a novel negative regulator of Type-I interferon signaling
Source: Sci Rep. 2016 Oct 26;6:36179. doi: 10.1038/srep36179 (PMC5080589; doi:10.1038/srep36179)
Supplement: Supplementary Information [file srep36179-s1.pdf]

Global functional profiling of human ubiquitome identifies E3 ubiquitin ligase DCST1 as a novel negative regulator of Type-I interferon signaling

Sajith Nair, Pradeep Bist, Neha Dikshit, Manoj Krishnan

Program in Emerging Infectious Diseases, Duke-NUS Medical School, Singapore 169857

SUPPLEMENTARY FIGURE 1. Uncropped images for all the panels shown in Figures 4B, 4F and 4G.

FIGURE 4B

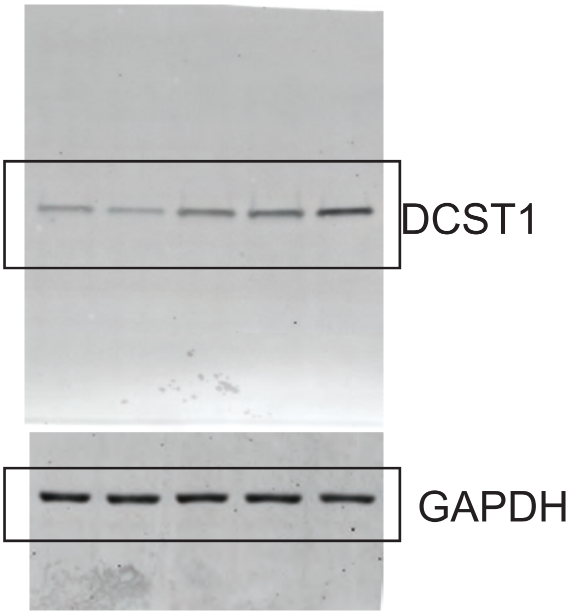

FIGURE 4F

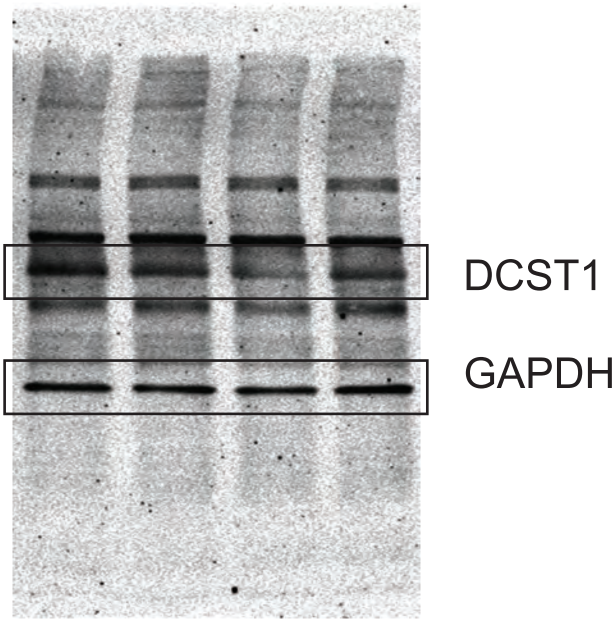

FIGURE 4G

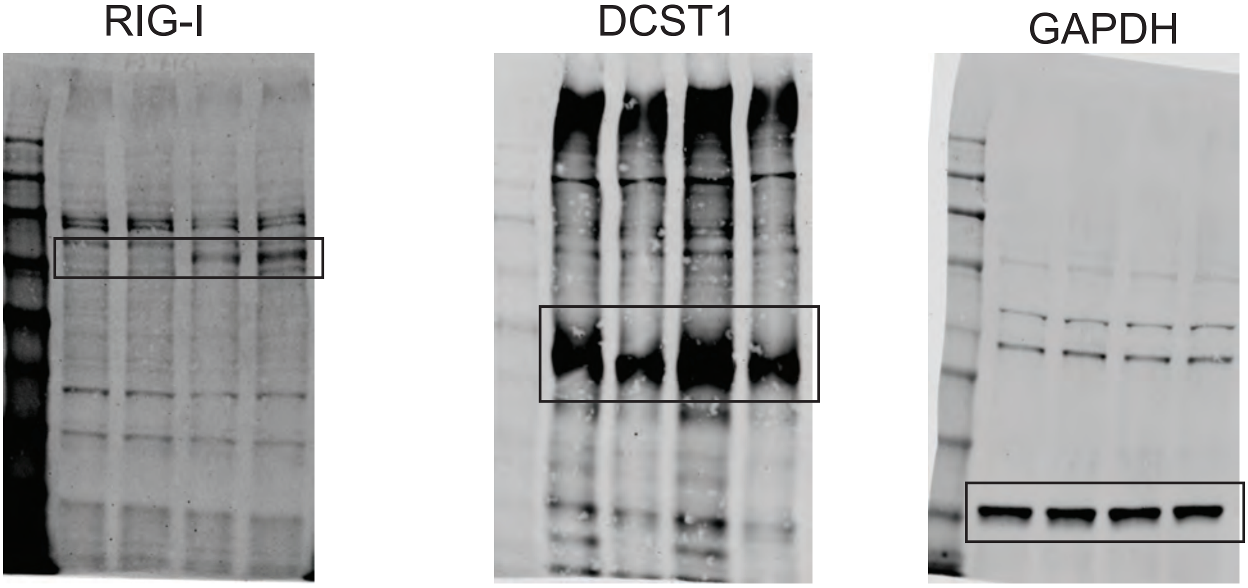

SUPPLEMENTARY FIGURE 2. Uncropped images for all the panels shown in Figure 5A.

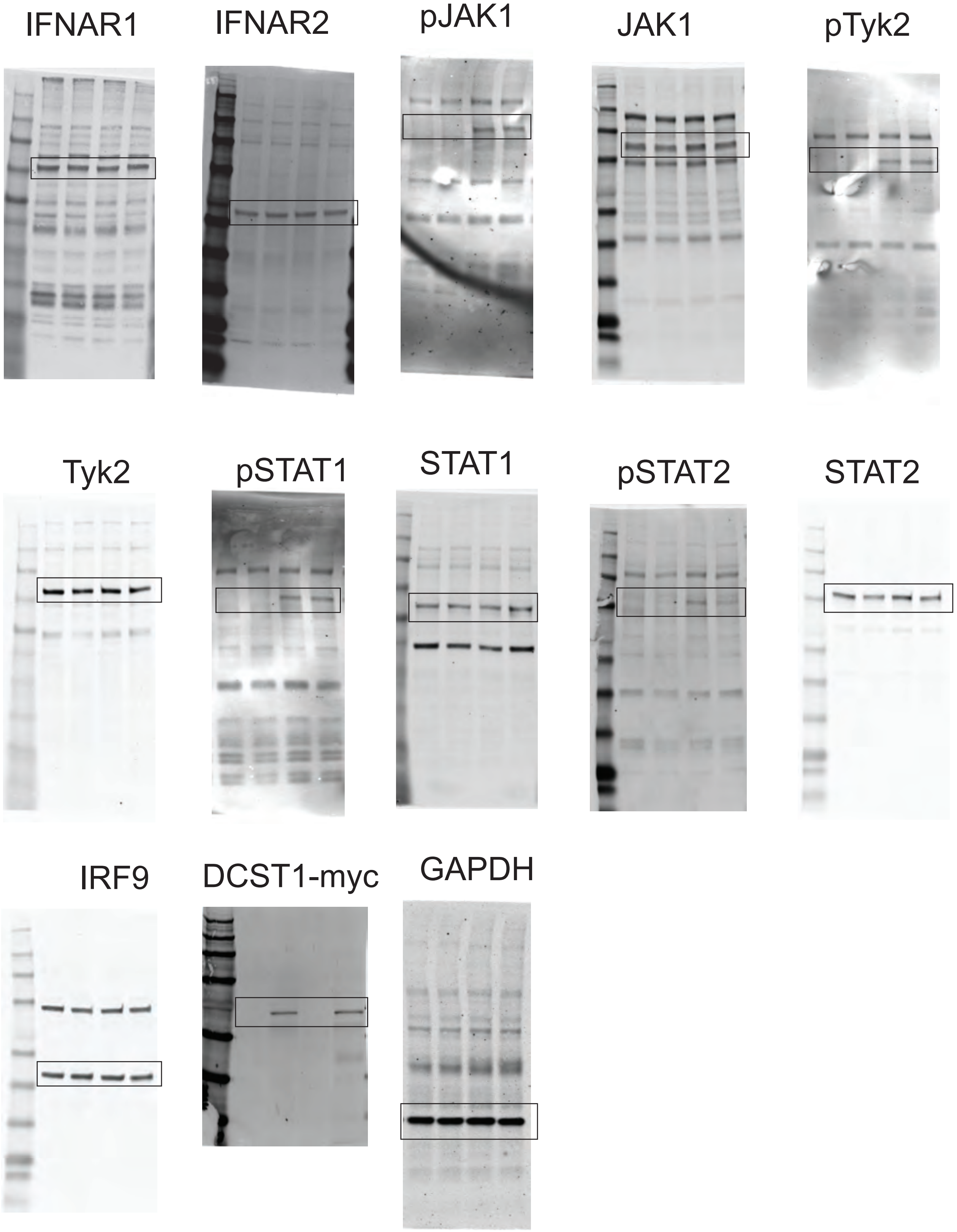

SUPPLEMENTARY FIGURE 3. Uncropped images for all the panels shown in Figure 5B.

IP: DCST1-HALO

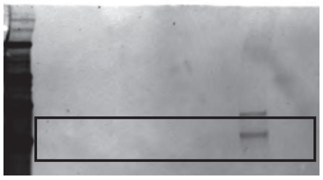

IP: GST

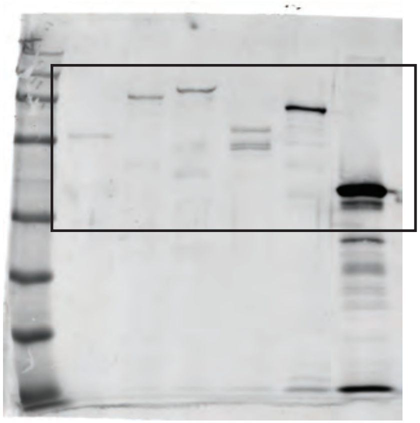

WCL: DCST1-HALO

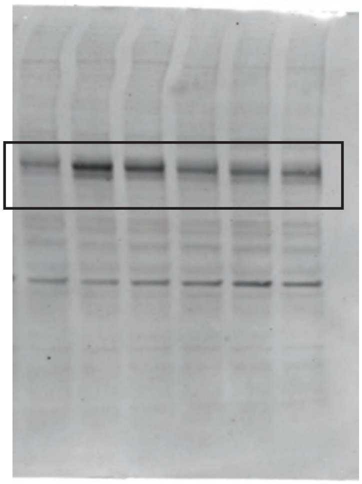

WCL: GST

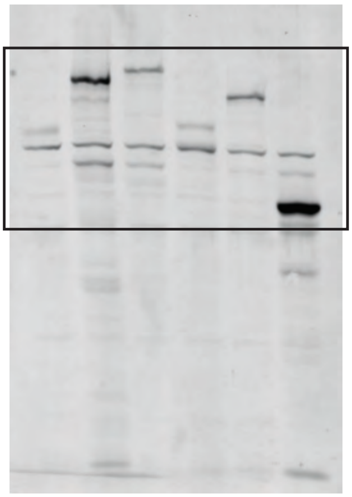

WCL: GAPDH

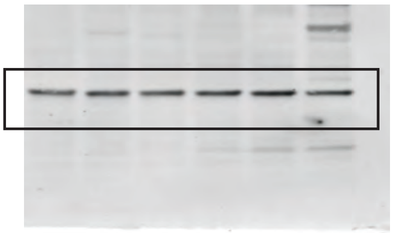

SUPPLEMENTARY FIGURE 4. Uncropped images for all the panels shown in Figure 5C.

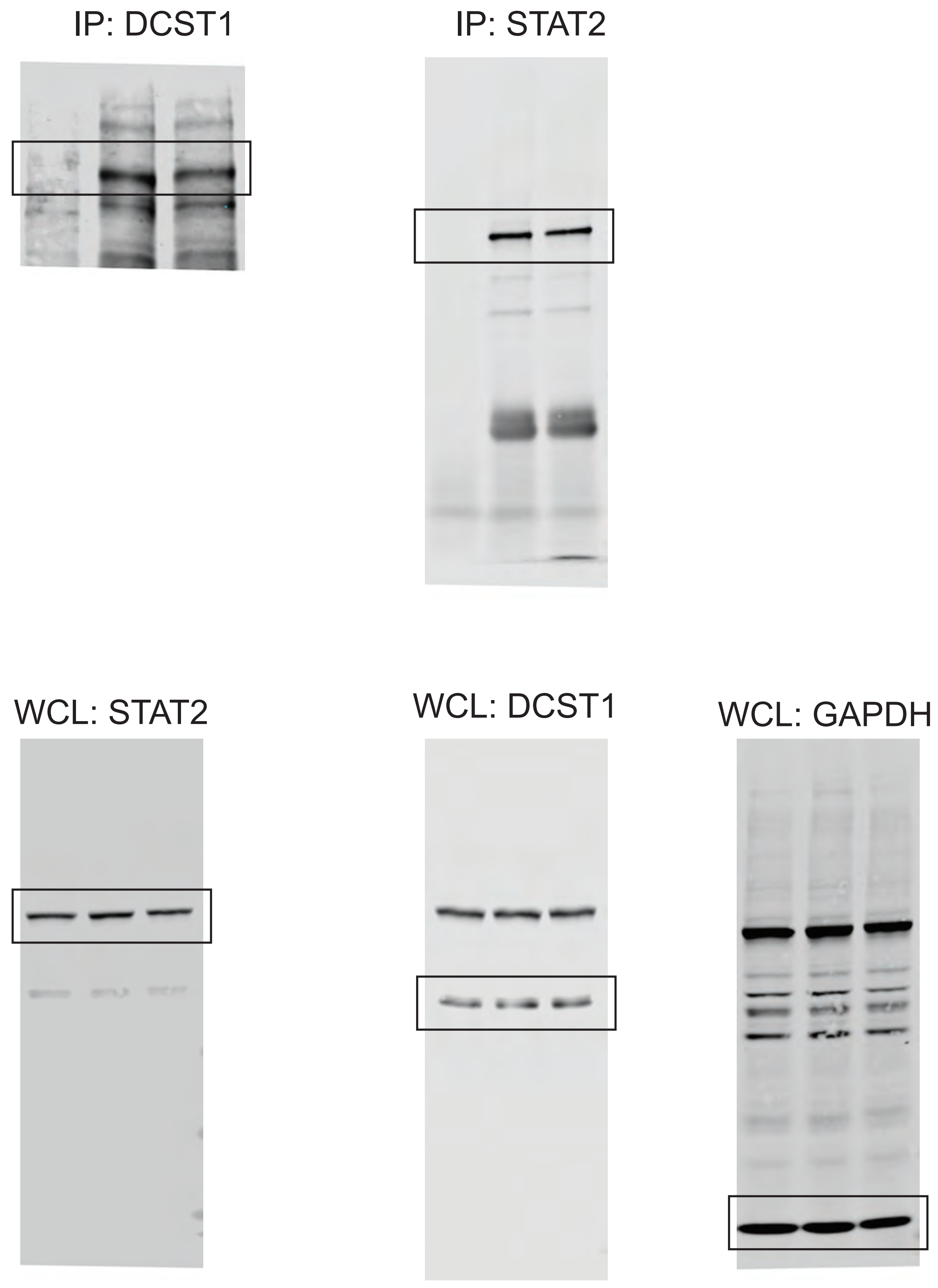

SUPPLEMENTARY FIGURE 5. Uncropped images for all the panels shown in Figure 5D

IP: DCST1-myc

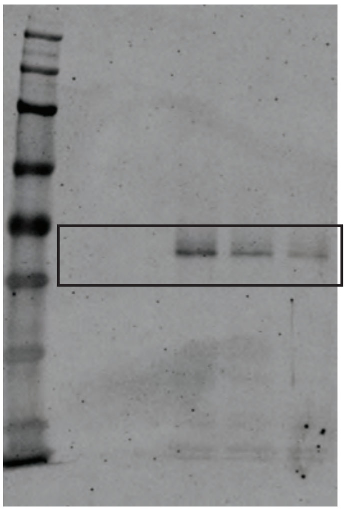

IP: GST

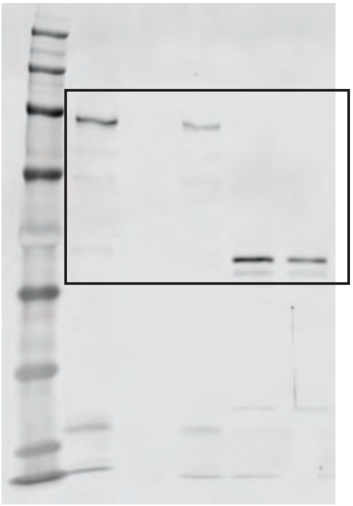

WCL: DCST1-myc

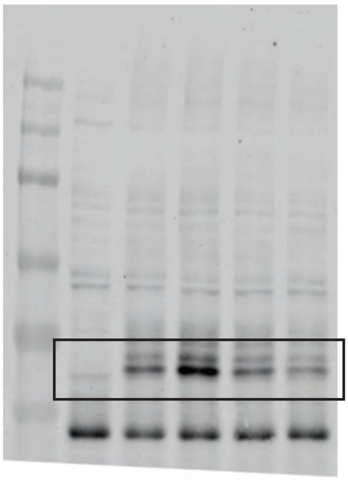

WCL: GST

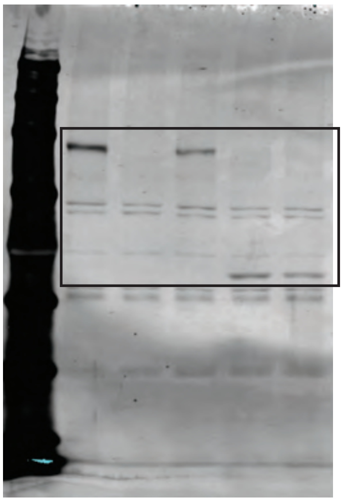

WCL: GAPDH

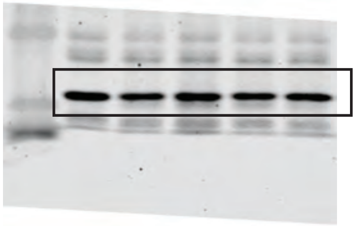

SUPPLEMENTARY FIGURE 6. Uncropped images for all the panels shown in Figure 5E.

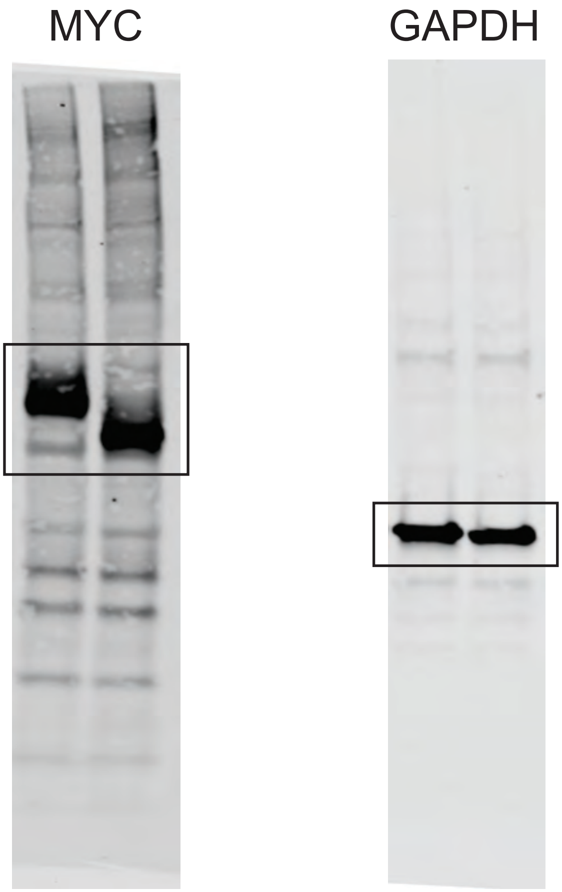

SUPPLEMENTARY FIGURE 7. Uncropped images for all the panels shown in Figure 5F.

DCST1-WT Panels

pSTAT2 STAT2 myc GAPDH

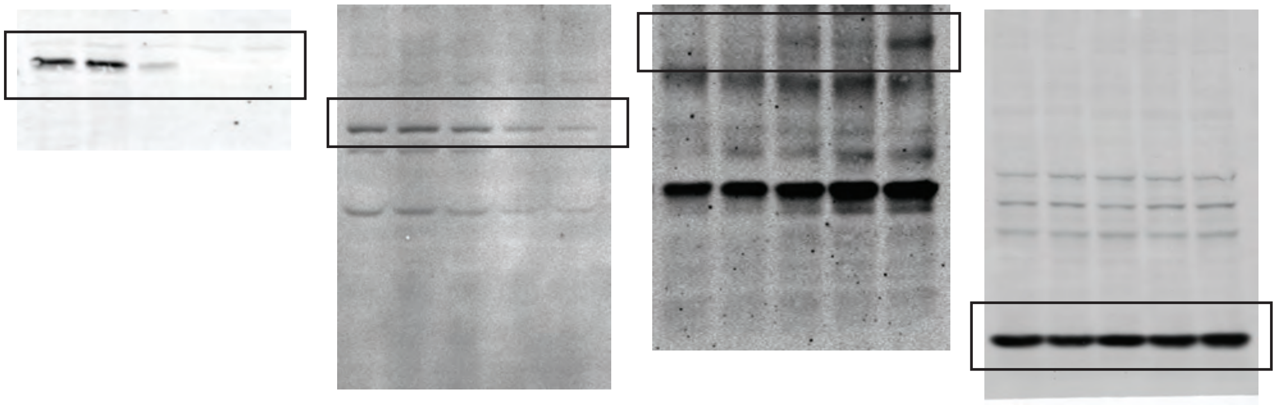

DCST1-RING Deletion Mutant Panels

pSTAT2 STAT2 myc GAPDH

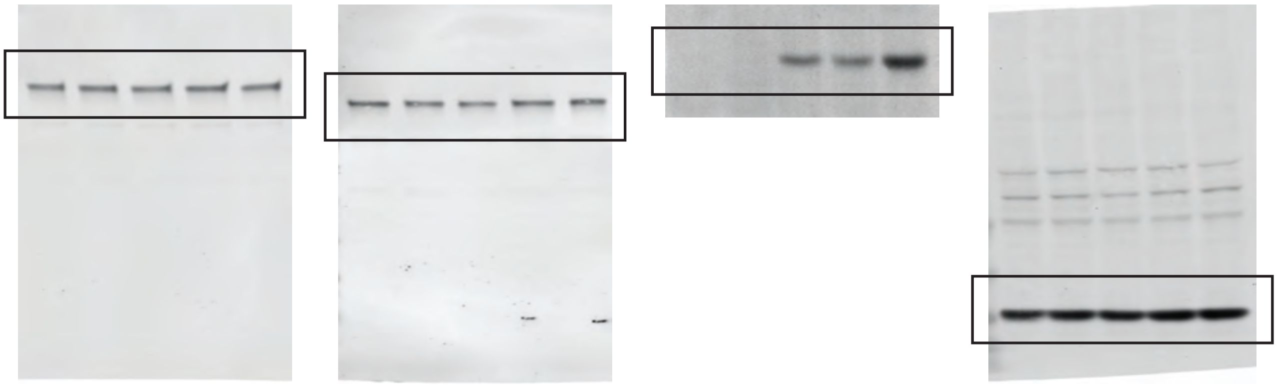

SUPPLEMENTARY FIGURE 8. Uncropped images for all the pnels shown in Figure 5G.

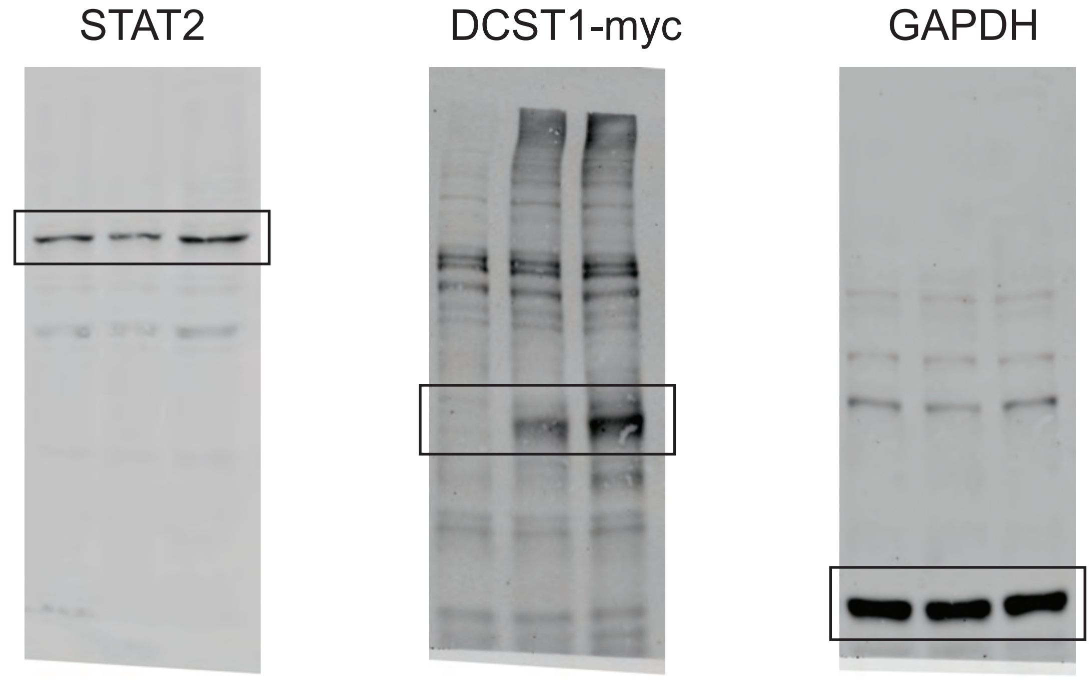

SUPPLEMENTARY FIGURE 9. Uncropped images for all the panels shown in Figure 6A.

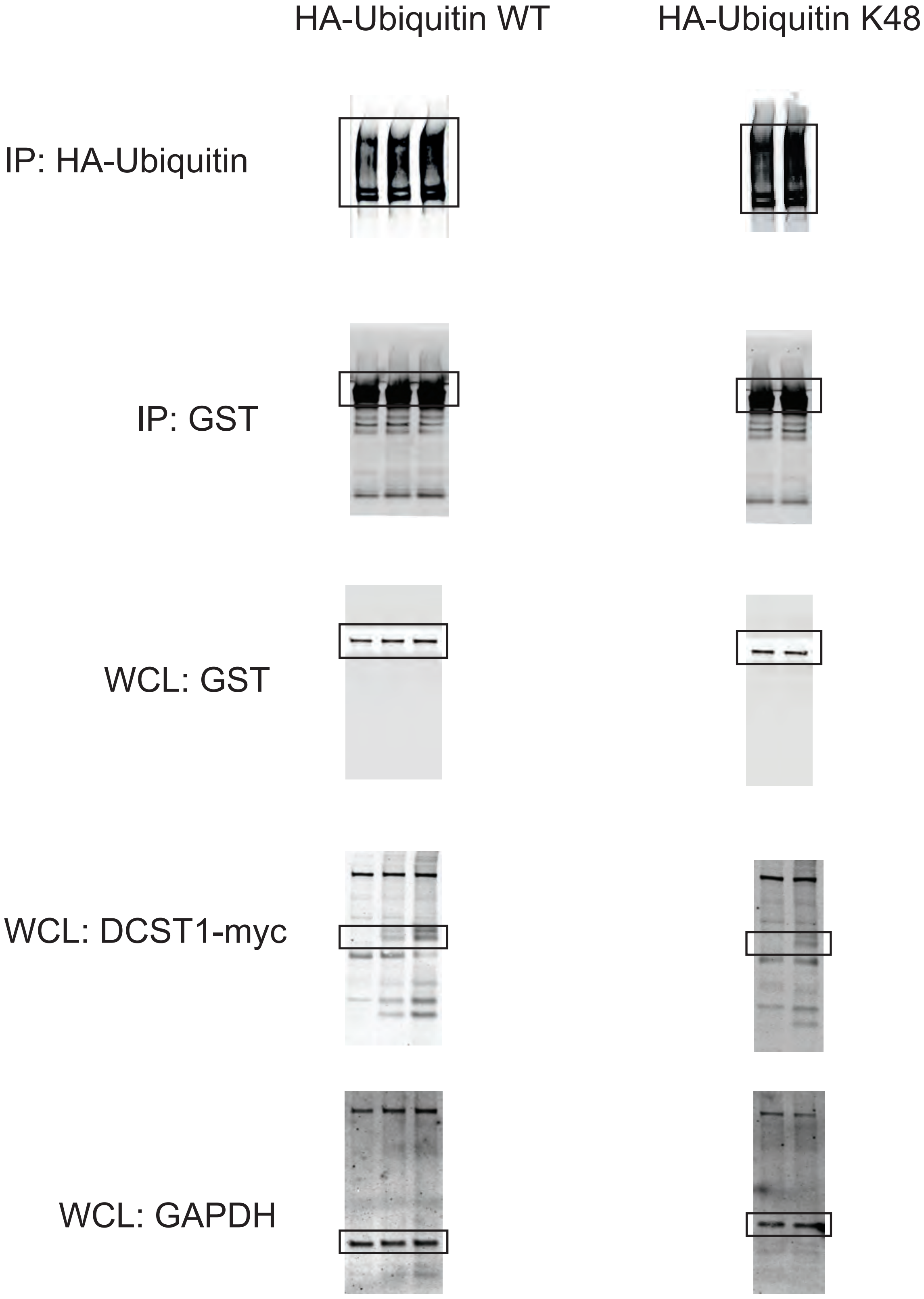

SUPPLEMENTARY FIGURE 10. Uncropped images for all the panels shown in Figure 6B.

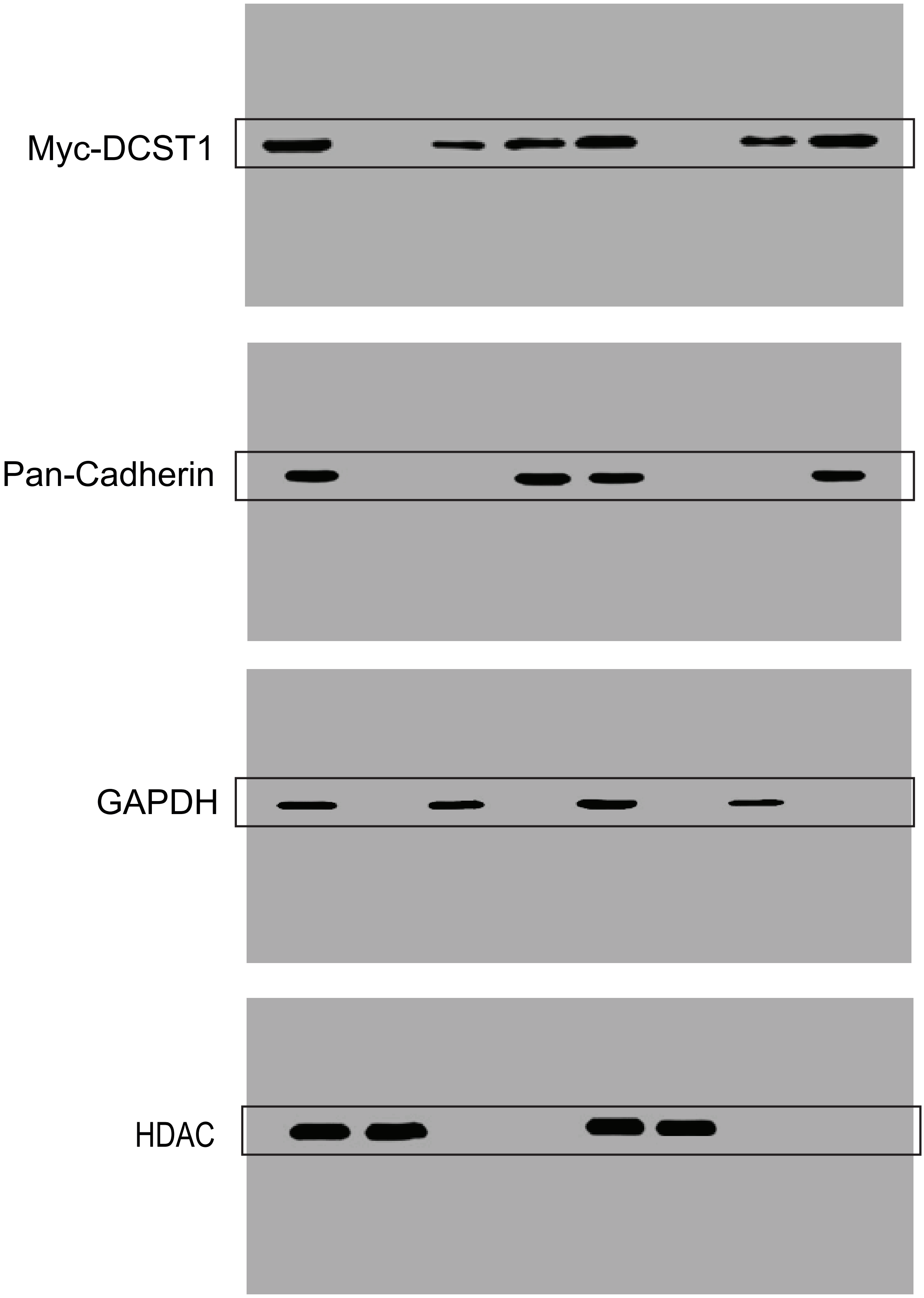

Global functional profiling of human ubiquitome identifies E3 ubiquitin ligase DCST1 as a novel negative regulator of Type-I interferon signaling

Sajith Nair, Pradeep Bist, Neha Dikshit and Manoj N Krishnan

Supplementary Table 1. **List of all tested human ubiquitome genes modulating IFN-I signaling**.
Column A=Gene Symbol; Column B= Gene ID; Column C = Gene Name; Column D = Accession Number.

	Gene Symbol	Gene ID	Accession Number
1	ABTB1	80325	NM_032548 NM_172027 NM_172028
2	ABTB2	25841	NM_145804
3	AMFR	267	NM_001144
4	ANAPC11	51529	NM_001002244 NM_001002245 NM_001002246 NM_001002247 NM_001002248 NM_001002249 NM_016476
5	ANKFY1	51479	NM_016376 NM_020740
6	ANKIB1	54467	NM_019004
7	ARIH1	25820	NM_005744
8	ARIH2	10425	NM_006321
9	ARMC5	79798	NM_024742
10	ASB10	136371	NM_080871
11	ASB11	140456	NM_001012428 NM_080873
12	ASB13	79754	NM_024701
13	ASB14	142686	NM_130387
14	ASB15	142685	NM_080928
15	ASB16	92591	NM_080863
16	ASB17	127247	NM_080868
17	ASB2	51676	NM_016150
18	ASB3	51130	NM_016115 NM_145863
19	ASB4	51666	NM_016116 NM_145872
20	ASB5	140458	NM_080874
21	ASB6	140459	NM_017873 NM_177999
22	ASB7	140460	NM_024708 NM_198243
23	ASB8	140461	NM_024095
24	ASB9	140462	NM_001031739 NM_024087
25	ATRX	546	NM_138271 NM_000489 NM_138270
26	BACH1	571	NM_001011545 NM_001186 NM_206866
27	BACH2	60468	NM_021813
28	BARD1	580	NM_000465
29	BCL6	604	NM_001706 NM_138931
30	BCL6B	255877	NM_181844
31	BFAR	51283	NM_016561
32	BIRC2	329	NM_001166
33	BIRC3	330	NM_001165 NM_182962
34	BIRC4	331	NM_001167
35	BIRC7	79444	NM_022161 NM_139317
36	BIRC8	112401	NM_033341
37	BMI1	648	NM_005180
38	BRAP	8315	NM_006768
39	BRCA1	672	NM_007294 NM_007295 NM_007296 NM_007297 NM_007298 NM_007299 NM_007300 NM_007302 NM_007303 NM_007304 NM_007305
40	BTBD1	53339	NM_001011885 NM_025238
41	BTBD10	84280	NM_032320
42	BTBD12	84464	NM_032444
43	BTBD14B	112939	NM_052876

44	BTBD2	55643	NM_017797
45	BTBD3	22903	NM_014962 NM_181443
46	BTBD7	55727	NM_001002860 NM_018167
47	BTBD9	114781	NM_152733
48	BTRC	8945	NM_003939 NM_033637
49	C13orf7	79596	NM_024546
50	C1orf164	55182	NM_018150
51	C1orf166	79594	NM_024544
52	CBL	867	NM_005188
53	CBLB	868	NM_170662
54	CBLC	23624	NM_012116
55	CBLL1	79872	NM_024814
56	CCIN	881	NM_005893
57	CCNB1IP1	57820	NM_021178 NM_182849 NM_182851 NM_182852
58	CCNF	899	NM_001761
59	CGRRF1	10668	NM_006568
60	CHFR	55743	NM_018223
61	CISH	1154	NM_145071
62	CNOT4	4850	NM_001008225 NM_013316
63	CPSF1	29894	NM_013291
64	DCST1	149095	NM_152494
65	DDB1	1642	NM_001923
66	DPF1	8193	NM_004647
67	DTX1	1840	NM_004416
68	DTX2	113878	NM_020892
69	DTX3	196403	NM_178502
70	DTX3L	151636	NM_138287
71	DTX4	23220	NM_015177
72	DZIP3	9666	NM_014648
73	ENC1	8507	NM_003633
74	FANCL	55120	NM_018062
75	FBXL12	54850	NM_017703
76	FBXL13	222235	NM_145032
77	FBXL14	144699	NM_152441
78	FBXL15	79176	NM_024326
79	FBXL16	146330	NM_153350
80	FBXL18	80028	NM_024963
81	FBXL2	25827	NM_012157
82	FBXL20	84961	NM_032875
83	FBXL3	26224	NM_012158
84	FBXL4	26235	NM_012160
85	FBXL5	26234	NM_012161 NM_033535
86	FBXL6	26233	NM_012162 NM_024555
87	FBXL7	23194	NM_012304
88	FBXL8	55336	NM_018378
89	FBXO11	80204	NM_012167 NM_018693 NM_025133
90	FBXO15	201456	NM_152676
91	FBXO17	115290	NM_024907 NM_148169
92	FBXO18	84893	NM_032807 NM_178150
93	FBXO2	26232	NM_012168
94	FBXO21	23014	NM_015002 NM_033624
95	FBXO22	26263	NM_012170 NM_147188
96	FBXO24	26261	NM_012172 NM_033506
97	FBXO25	26260	NM_012173 NM_183420 NM_183421
98	FBXO27	126433	NM_178820
99	FBXO28	23219	NM_015176
100	FBXO3	26273	NM_012175 NM_033406
101	FBXO30	84085	NM_032145
102	FBXO32	114907	NM_058229 NM_148177
103	FBXO33	254170	NM_203301
104	FBXO34	55030	NM_017943
105	FBXO36	130888	NM_174899
106	FBXO38	81545	NM_030793 NM_205836
107	FBXO4	26272	NM_012176 NM_033484
108	FBXO40	51725	NM_016298

109	FBXO42	54455	NM_018994
110	FBXO44	93611	NM_001014765 NM_033182 NM_183412 NM_183413
111	FBXO45	200933	XM_931557 XM_946180
112	FBXO46	23403	NM_001080469
113	FBXO5	26271	NM_012177
114	FBXO6	26270	NM_018438
115	FBXO7	25793	NM_001033024 NM_012179
116	FBXO8	26269	NM_012180
117	FBXO9	26268	NM_012347 NM_033480 NM_033481
118	FBXW11	23291	NM_012300 NM_033644 NM_033645
119	FBXW2	26190	NM_012164
120	FBXW4	6468	NM_022039
121	FBXW5	54461	NM_018998
122	FBXW7	55294	NM_001013415 NM_018315 NM_033632
123	FBXW8	26259	NM_012174 NM_153348
124	FLJ30092	196515	XM_497354 XM_942665
125	GAN	8139	NM_022041
126	GMCL1	64395	NM_178439
127	GTF2H2	2966	NM_001515
128	GZF1	64412	NM_022482
129	HACE1	57531	NM_020771
130	HECTD2	143279	NM_173497 NM_182765
131	HECTD3	79654	NM_024602
132	HECW1	23072	NM_015052
133	HERC3	8916	NM_014606
134	HERC4	26091	NM_001017972 NM_015601 NM_022079
135	HERC6	55008	NM_001013000 NM_001013002 NM_001013005 NM_017912
136	HIC1	3090	NM_001098202 NM_006497
137	HIC2	23119	NM_015094
138	HLTF	6596	NM_003071 NM_139048
139	IBRDC1	154214	NM_152553
140	IBRDC2	255488	NM_182757
141	IBRDC3	127544	NM_153341
142	IBTK	25998	NM_015525
143	ITCH	83737	NM_031483
144	IVNS1ABP	10625	NM_016389 NM_006469
145	KBTBD10	10324	NM_006063
146	KBTBD2	25948	NM_015483
147	KBTBD3	143879	NM_152433 NM_198439
148	KBTBD4	55709	NM_016506 NM_018095
149	KBTBD5	131377	NM_152393
150	KBTBD6	89890	NM_152903
151	KBTBD7	84078	NM_032138
152	KBTBD8	84541	NM_032505
153	KCNA2	3737	NM_004974
154	KCNA3	3738	NM_002232
155	KCNA5	3741	NM_002234
156	KCNA6	3742	NM_002235
157	KCNC1	3746	NM_004976
158	KCND1	3750	NM_004979
159	KCND2	3751	NM_012281
160	KCND3	3752	NM_004980 NM_172198
161	KCNG1	3755	NM_002237 NM_172318
162	KCNG3	170850	NM_133329 NM_172344
163	KCNRG	283518	NM_173605 NM_199464
164	KCNS2	3788	NM_020697
165	KCNS3	3790	NM_002252
166	KCTD1	284252	NM_198991
167	KCTD10	83892	NM_031954
168	KCTD12	115207	NM_138444
169	KCTD13	253980	NM_178863
170	KCTD14	65987	NM_023930
171	KCTD15	79047	NM_024076
172	KCTD16	57528	NM_020768

173	KCTD17	79734	NM_024681
174	KCTD20	222658	NM_173562
175	KCTD3	51133	NM_016121
176	KCTD4	386618	NM_198404
177	KCTD5	54442	NM_018992
178	KCTD6	200845	NM_153331
179	KCTD8	386617	NM_198353
180	KCTD9	54793	NM_017634
181	KEAP1	9817	NM_012289 NM_203500
182	KIAA0317	9870	NM_001039479
183	KIAA1333	55632	NM_017769
184	KLHDC5	57542	NM_020782
185	KLHL1	57626	NM_020866
186	KLHL10	317719	NM_152467
187	KLHL11	55175	NM_018143
188	KLHL12	59349	NM_021633
189	KLHL13	90293	NM_033495
190	KLHL14	57565	NM_020805
191	KLHL15	80311	NM_030624
192	KLHL17	339451	NM_198317
193	KLHL18	23276	NM_025010
194	KLHL20	27252	NM_014458
195	KLHL21	9903	NM_014851
196	KLHL22	84861	NM_032775
197	KLHL23	151230	NM_144711
198	KLHL24	54800	NM_017644
199	KLHL25	64410	NM_022480
200	KLHL26	55295	NM_018316
201	KLHL28	54813	NM_017658
202	KLHL29	114818	XM_001134449 XM_940375
203	KLHL3	26249	NM_017415
204	KLHL30	377007	NM_198582
205	KLHL31	401265	NM_001003760
206	KLHL32	114792	NM_052904
207	KLHL34	257240	NM_153270
208	KLHL6	89857	NM_130446
209	KLHL7	55975	NM_001031710 NM_018846
210	KLHL8	57563	NM_020803
211	KLHL9	55958	NM_018847
212	LGALS3BP	3959	NM_005567
213	LINCR	93082	NM_001080535
214	LNX1	84708	NM_032622
215	LNX2	222484	NM_153371
216	LONRF1	91694	NM_152271
217	LONRF3	79836	NM_001031855 NM_024778
218	LRRC29	26231	NM_001004055 NM_012163
219	LRSAM1	90678	NM_001005373 NM_001005374 NM_138361
220	LZTR1	8216	NM_006767
221	'MARCH1	55016	NM_017923
222	'MARCH2	51257	NM_001005415 NM_001005416 NM_016496
223	'MARCH3	115123	NM_178450
224	'MARCH8	220972	NM_001002265 NM_001002266 NM_145021
225	'MARCH9	92979	NM_138396
226	MDM2	4193	NM_002392 NM_006878 NM_006879 NM_006881 NM_006882
227	MDM4	4194	NM_002393
228	MGC23270	196872	NM_152646
229	MGRN1	23295	NM_015246
230	MIB1	57534	NM_020774
231	MIB2	142678	NM_080875
232	MID1	4281	NM_033291 NM_000381 NM_001098624 NM_033290
233	MID2	11043	NM_012216 NM_052817
234	MKRN1	23608	NM_013446
235	MKRN2	23609	NM_014160
236	MKRN3	7681	NM_005664

237	MNAT1	4331	NM_002431
238	MYLIP	29116	NM_013262
239	MYNN	55892	NM_018657
240	NEDD4	4734	NM_006154 NM_198400
241	NEDD4L	23327	NM_015277
242	NEURL	9148	NM_004210
243	NEURL2	140825	NM_080749
244	NFX1	4799	NM_002504 NM_147133 NM_147134
245	NHLRC1	378884	NM_198586
246	NOSIP	51070	NM_015953
247	NSMCE1	197370	NM_145080
248	OSTM1	28962	NM_014028
249	OTUD7B	56957	NM_020205
250	PARK2	5071	NM_004562 NM_013987 NM_013988
251	PATZ1	23598	NM_014323 NM_032050 NM_032051 NM_032052
252	PCGF1	84759	NM_032673
253	PCGF2	7703	NM_007144
254	PCGF3	10336	NM_006315
255	PCGF5	84333	NM_032373
256	PCGF6	84108	NM_001011663 NM_032154
257	PDZRN3	23024	NM_015009
258	PEX10	5192	NM_002617 NM_153818
259	PEX12	5193	NM_000286
260	PHF7	51533	NM_016483 NM_173341
261	PJA1	64219	NM_001032396 NM_022368 NM_145119
262	PJA2	9867	NM_014819
263	PML	5371	NM_002675 NM_033238 NM_033239 NM_033240 NM_033244 NM_033246 NM_033247 NM_033249 NM_033250
264	PPIL2	23759	NM_014337 NM_148175 NM_148176
265	PRPF19	27339	NM_014502
266	PXMP3	5828	NM_000318 NM_001079867
267	RAB40A	142684	NM_080879
268	RAB40B	10966	NM_006822
269	RAB40C	57799	NM_021168
270	RABGEF1	27342	NM_014504
271	RAD18	56852	NM_020165
272	RAG1	5896	NM_000448
273	RAPSN	5913	NM_005055 NM_032645
274	RBBP6	5930	NM_006910 NM_018703 NM_032626
275	RBCK1	10616	NM_006462 NM_031229
276	RBX1	9978	NM_014248
277	RC3H1	149041	NM_172071
278	RC3H2	54542	NM_018835
279	RCBTB1	55213	NM_018191
280	RCBTB2	1102	NM_001268
281	RCHY1	25898	NM_001008925 NM_001009922 NM_015436
282	RFFL	117584	NM_001017368 NM_057178
283	RFPL1	5988	NM_021026
284	RFPL3	10738	NM_001098535 NM_006604
285	RFPL4A	342931	XM_292796
286	RFPL4B	442247	NM_001013734
287	RFWD2	64326	NM_001001740 NM_022457
288	RFWD3	55159	NM_018124
289	RHOBTB1	9886	NM_001032380 NM_014836 NM_198225
290	RHOBTB3	22836	NM_014899
291	RING1	6015	NM_002931
292	RNF10	9921	NM_014868
293	RNF103	7844	NM_005667
294	RNF11	26994	NM_014372
295	RNF111	54778	NM_017610
296	RNF113A	7737	NM_006978
297	RNF113B	140432	NM_178861
298	RNF121	55298	NM_018320 NM_194452 NM_194453
299	RNF122	79845	NM_024787

300	RNF125	54941	NM_017831
301	RNF126	55658	NM_017876 NM_194460
302	RNF128	79589	NM_024539 NM_194463
303	RNF13	11342	NM_183382 NM_007282 NM_183381 NM_183383 NM_183384
304	RNF130	55819	NM_018434
305	RNF133	168433	NM_139175
306	RNF135	84282	NM_032322 NM_197939
307	RNF138	51444	NM_016271 NM_198128
308	RNF139	11236	NM_007218
309	RNF14	9604	NM_004290 NM_183398 NM_183399 NM_183400 NM_183401
310	RNF141	50862	NM_016422
311	RNF144	9781	NM_014746
312	RNF145	153830	NM_144726
313	RNF146	81847	NM_030963
314	RNF148	378925	NM_198085
315	RNF150	57484	NM_020724
316	RNF151	146310	NM_174903
317	RNF152	220441	NM_173557
318	RNF157	114804	NM_052916
319	RNF165	494470	NM_152470
320	RNF166	115992	NM_178841
321	RNF167	26001	NM_015528
322	RNF168	165918	NM_152617
323	RNF169	254225	NM_001098638
324	RNF170	81790	NM_030954
325	RNF175	285533	NM_173662
326	RNF180	285671	NM_178532
327	RNF181	51255	NM_016494
328	RNF182	221687	NM_152737
329	RNF183	138065	NM_145051
330	RNF185	91445	NM_152267
331	RNF186	54546	NM_019062
332	RNF187	149603	XM_001129309 XM_928029
333	RNF2	6045	NM_007212
334	RNF20	56254	NM_019592
335	RNF207	388591	NM_173795 NM_207396
336	RNF212	285498	NM_194439
337	RNF213	57674	NM_020914
338	RNF214	257160	NM_001077239 NM_207343
339	RNF215	200312	NM_001017981
340	RNF24	11237	NM_007219
341	RNF25	64320	NM_022453
342	RNF26	79102	NM_032015
343	RNF31	55072	NM_017999
344	RNF32	140545	NM_030936
345	RNF34	80196	NM_025126 NM_194271
346	RNF38	152006	NM_022781 NM_194328 NM_194329 NM_194330 NM_194332
347	RNF39	80352	NM_170770 NM_025236 NM_170769
348	RNF4	6047	NM_002938
349	RNF40	9810	NM_014771
350	RNF41	10193	NM_005785 NM_194358 NM_194359
351	RNF43	54894	NM_017763
352	RNF44	22838	NM_014901
353	RNF5	6048	NM_006913
354	RNF6	6049	NM_183045 NM_005977 NM_183043 NM_183044
355	RNF7	9616	NM_014245 NM_183237
356	RNF8	9025	NM_003958 NM_183078
357	RSPRY1	89970	NM_133368
358	SF3B3	23450	NM_012426
359	SH3RF1	57630	NM_020870
360	SH3RF2	153769	NM_152550
361	SHKBP1	92799	NM_138392

362	SHPRH	257218	NM_001042683 NM_173082
363	SIAH1	6477	NM_001006610 NM_003031
364	SIAH2	6478	NM_005067
365	SKP2	6502	NM_005983 NM_032637
366	SMURF1	57154	NM_020429 NM_181349
367	SMURF2	64750	NM_022739
368	SOCS1	8651	NM_003745
369	SOCS2	8835	NM_003877
370	SOCS3	9021	NM_003955
371	SOCS4	122809	NM_080867 NM_199421
372	SOCS5	9655	NM_014011 NM_144949
373	SOCS6	9306	NM_004232
374	SPOP	8405	NM_001007226 NM_001007227 NM_001007228 NM_001007229 NM_001007230 NM_003563
375	SPRYD5	84767	NM_032681
376	SPSB1	80176	NM_025106
377	SPSB2	84727	NM_032641
378	SPSB3	90864	NM_080861
379	SPSB4	92369	NM_080862
380	STUB1	10273	NM_005861
381	SYVN1	84447	NM_032431 NM_172230
382	TCEB3	6924	NM_003198
383	TNFAIP1	7126	NM_021137
384	TNFAIP3	7128	NM_006290
385	TRAF2	7186	NM_021138
386	TRAF3	7187	NM_003300 NM_145725 NM_145726
387	TRAF4	9618	NM_145751 NM_004295
388	TRAF5	7188	NM_001033910 NM_004619 NM_145759
389	TRAF6	7189	NM_004620 NM_145803
390	TRAF7	84231	NM_206835 NM_032271
391	TRAIP	10293	NM_005879
392	TRIAD3	54476	NM_207111 NM_207116
393	TRIM10	10107	NM_006778 NM_052828
394	TRIM11	81559	NM_145214
395	TRIM13	10206	NM_001007278 NM_005798 NM_052811 NM_213590
396	TRIM15	89870	NM_052812 NM_033229
397	TRIM17	51127	NM_001024940 NM_001024941 NM_016102
398	TRIM2	23321	NM_015271
399	TRIM21	6737	NM_003141
400	TRIM22	10346	NM_006074
401	TRIM23	373	NM_001656 NM_033227 NM_033228
402	TRIM24	8805	NM_003852 NM_015905
403	TRIM25	7706	NM_005082
404	TRIM26	7726	NM_003449
405	TRIM27	5987	NM_030950 NM_006510
406	TRIM28	10155	NM_005762
407	TRIM3	10612	NM_006458 NM_033278
408	TRIM31	11074	NM_052816 NM_007028
409	TRIM32	22954	NM_012210
410	TRIM33	51592	NM_015906 NM_033020
411	TRIM34	53840	NM_001003827 NM_021616 NM_130389 NM_130390
412	TRIM35	23087	NM_171982
413	TRIM36	55521	NM_001017397 NM_001017398 NM_018700
414	TRIM37	4591	NM_001005207 NM_015294
415	TRIM38	10475	NM_006355
416	TRIM39	56658	NM_021253 NM_172016
417	TRIM4	89122	NM_033017 NM_033091
418	TRIM40	135644	NM_138700
419	TRIM41	90933	NM_033549 NM_201627
420	TRIM42	287015	NM_152616
421	TRIM43	129868	NM_138800
422	TRIM45	80263	NM_025188
423	TRIM46	80128	NM_025058
424	TRIM47	91107	NM_033452
425	TRIM48	79097	NM_024114

426	TRIM49	57093	NM_020358
427	TRIM5	85363	NM_033034 NM_033092 NM_033093
428	TRIM50	135892	NM_178125
429	TRIM52	84851	NM_032765
430	TRIM54	57159	NM_032546 NM_187841
431	TRIM55	84675	NM_033058 NM_184085 NM_184086 NM_184087
432	TRIM56	81844	NM_030961
433	TRIM58	25893	NM_015431
434	TRIM59	286827	NM_173084
435	TRIM6	117854	NM_001003818 NM_058166
436	TRIM60	166655	NM_152620
437	TRIM61	391712	NM_001012414
438	TRIM62	55223	NM_018207
439	TRIM63	84676	NM_032588
440	TRIM65	201292	NM_173547
441	TRIM67	440730	NM_001004342
442	TRIM68	55128	NM_018073
443	TRIM69	140691	NM_080745 NM_182985
444	TRIM7	81786	NM_033342 NM_203293 NM_203294 NM_203295 NM_203296 NM_203297
445	TRIM72	493829	NM_001008274
446	TRIM73	375593	NM_198924
447	TRIM74	378108	NM_198853
448	TRIM8	81603	NM_030912
449	TRIM9	114088	NM_015163 NM_052978
450	TRIML1	339976	NM_178556
451	TRIP12	9320	NM_004238
452	TTC3	7267	NM_001001894 NM_003316
453	TULP4	56995	NM_001007466 NM_020245
454	UBE3A	7337	NM_000462 NM_130838 NM_130839
455	UBE3B	89910	NM_130466 NM_183415
456	UBE3C	9690	NM_014671
457	UBE4A	9354	NM_004788
458	UBE4B	10277	NM_006048
459	UBOX5	22888	NM_014948 NM_199415
460	UBR2	23304	NM_015255
461	UHRF1	29128	NM_001048201 NM_013282
462	UHRF2	115426	NM_152896
463	UNK	85451	NM_001080419
464	VHL	7428	NM_000551 NM_198156
465	VPS11	55823	NM_021729
466	VPS18	57617	NM_020857
467	VPS41	27072	NM_014396 NM_080631
468	VPS8	23355	NM_001009921 NM_015303
469	WDSUB1	151525	NM_152528
470	WHSC1	7468	NM_133336 NM_001042424 NM_007331 NM_133330 NM_133331 NM_133334 NM_133335
471	WSB1	26118	NM_015626 NM_134265
472	WSB2	55884	NM_018639
473	WWP1	11059	NM_007013
474	WWP2	11060	NM_007014 NM_199423 NM_199424
475	ZBTB1	22890	NM_014950
476	ZBTB10	65986	NM_023929
477	ZBTB11	27107	NM_014415
478	ZBTB16	7704	NM_001018011 NM_006006
479	ZBTB17	7709	NM_003443
480	ZBTB2	57621	NM_020861
481	ZBTB20	26137	NM_015642
482	ZBTB22	9278	NM_005453
483	ZBTB24	9841	NM_014797
484	ZBTB25	7597	NM_006977
485	ZBTB26	57684	NM_020924
486	ZBTB3	79842	NM_024784
487	ZBTB32	27033	NM_014383
488	ZBTB33	10009	NM_006777

489	ZBTB37	84614	NM_032522
490	ZBTB38	253461	NM_001080412
491	ZBTB39	9880	NM_014830
492	ZBTB4	57659	NM_020899
493	ZBTB40	9923	NM_001083621 NM_014870
494	ZBTB41	360023	NM_194314
495	ZBTB43	23099	NM_014007
496	ZBTB44	29068	NM_014155
497	ZBTB45	84878	NM_032792
498	ZBTB46	140685	NM_025224
499	ZBTB48	3104	NM_005341
500	ZBTB5	9925	NM_014872
501	ZBTB6	10773	NM_006626
502	ZBTB7A	51341	NM_015898
503	ZBTB7B	51043	NM_015872
504	ZBTB7C	201501	NM_001039360
505	ZBTB9	221504	NM_152735
506	ZFAND3	60685	NM_021943
507	ZFAND5	7763	NM_006007
508	ZFAND6	54469	NM_019006
509	ZFP161	7541	NM_003409
510	ZFPL1	7542	NM_006782
511	ZNF131	7690	NM_003432
512	ZNF238	10472	NM_006352 NM_205768
513	ZNF295	49854	NM_001098402 NM_001098403 NM_020727
514	ZNF313	55905	NM_018683
515	ZNF364	27246	NM_014455
516	ZNF598	90850	NM_178167
517	ZNF645	158506	NM_152577
518	ZNRF1	84937	NM_032268
519	ZNRF2	223082	NM_147128
520	ZNRF3	84133	NM_032173
521	ZSWIM2	151112	NM_182521

Global functional profiling of human ubiquitome identifies E3 ubiquitin ligase DCST1 as a novel negative regulator of Type-I interferon signaling

Sajith Nair, Pradeep Bist, Neha Dikshit and Manoj N Krishnan

Supplementary Table 2. List of all primers used for detection of gene expression using q-RTPCR. FP = forward primer; RP = reverse primer

CCIN	FP	AGGAGGATCGGGAGAAGTATT
CCIN	RP	GCAAACACCAGCGTCTTATTG
ASB16	FP	GCTCACTGCAACTTCTACCA
ASB16	RP	GGTGAGACCCTGTCTCTACTAA
(MARCH4)	FP	CTCTTCCTCATCGCCAGTATTT
(MARCH4)	RP	CACGTCCATGAAGCCATACA
RNF152	FP	TCTGGGTTGCCTCTTGATTAC
RNF152	RP	TGCCATCAACACTCAGAACA
KCNS2	FP	CTTTGCATCGTGGGCATAAA
KCNS2	RP	GGGCCACAAGCACTAGAATA
SOCS1	FP	CTTCTGTAGGATGGTAGCACAC
SOCS1	RP	AGGAAGAGGAGGAAGGTTCT
(MARCH9)	FP	CAGGGCATCTGGCAATAAGA
(MARCH9)	RP	GGAGTCTCCACGTGCATATAAA
TRIM7	FP	CCTGCGGAAGCATCTTGTA
TRIM7	RP	ACATGCCATGAGGCTCTTTAT
STUB1	FP	CAATCTGCAGCGAGCTTACA
STUB1	RP	CTGTTCCAGCGCTTCTTCTT
TCEB3	FP	CCACGGCACTTGGAGATAAA
TCEB3	RP	CGGCTTCTCTGACTTGGTTT
RNF44	FP	AGACTGAGCACCCAGAGATA
RNF44	RP	AGAGGAAGTAGGGCAGGAA
KEAP1	FP	CGTCCTGCACAACTGTATCT
KEAP1	RP	CGAAAGTCCACGTCTCTGTT
TRIM38	FP	TGATACTCTCCGGCCCTATT
TRIM38	RP	CGTCTGCCCTTATGGGATTT
MIB2	FP	GCAGGTGGACACCAAGAA
MIB2	RP	GTAGCAGCAGCCGTATCAA
DCST1	FP	TGGGCTCTCTACTCCATCTT
DCST1	RP	CGACCTTCACCTCCAGTTTAT
RNF175	FP	GCAGGAGAGGATGTACAAGATG

RNF175	RP	CAGCACTATCTGGGCAATGA
